# Multifunctional saikosaponin D-liposomes for hepatocellular carcinoma: Formulation optimization, characterization, and in vitro*/*in vivo evaluation

**DOI:** 10.1016/j.ijpx.2025.100445

**Published:** 2025-11-11

**Authors:** Kun Yu, Sicheng Huang, Guochun Yang, Junze Tang, Xiaoyu Zhao, Rui Pan, Hailiang Zhang, Menghan Xu, Xiaofei Li, Xin Cheng, Anguo Hou

**Affiliations:** a College of Traditional Chinese Medicine, Yunnan University of Chinese Medicine, Kunming 650500, China; b Yunnan Key Laboratory of Dai and Yi Medicines, Kunming 650500, China; c Laboratory Animal Center, Yunnan University of Chinese Medicine, Kunming 650500, China.; d The Key Laboratory of External Drug Delivery System and Preparation Technology in University of Yunnan Province, Kunming 650500, China

**Keywords:** Functional liposomes, Saikosaponin D, Hepatocellular carcinoma, Targeted drug delivery, Antitumor efficacy, SSD (CAS No.: 20874–52-6), P407 (CAS No.: 9003-11-6), SPC (CAS No.: 8002-43-5), PA (CAS No.: 383907–53-7), DOX (CAS No.: 25316–40-9), Chol (CAS No.: 57–88-5).

## Abstract

The study aimed to construct saikosaponin D (SSD)-based liposomes modified with phosphatidic acid (PA) and poloxamer 407 (P407) (termed P407-SSD-Lps), and to evaluate their tumor-targeting ability and antitumor efficacy through in vitro and in vivo experiments. The preparation process and formulation of the P407-SSD-Lps were optimized using single-factor and orthogonal experimental designs, followed by systematic characterization. Their antitumor activity and targeting specificity were assessed through in vitro experiments. Additionally, the tumor-targeting capability, therapeutic efficacy, and biocompatibility of the P407-SSD-Lps were investigated in murine orthotopic hepatocellular carcinoma transplantation models. The P407-SSD-Lps optimized through single-factor and orthogonal experiments exhibited ideal physicochemical properties. In vitro results demonstrated that the P407-SSD-Lps enhanced cell membrane permeability and promoted cellular uptake in the HepG2 cells. Additionally, they significantly inhibited the HepG2 cells proliferation and induced apoptosis. In murine orthotopic hepatocellular carcinoma transplantation models, the P407-SSD-Lps exhibited prolonged tumor accumulation and demonstrated potent antitumor efficacy with favorable biocompatibility. When delivering doxorubicin (DOX), the system not only retained high biocompatibility but also exhibited enhanced therapeutic efficacy. Employing the SSD as both a liposomal membrane stabilizer and a therapeutic agent constituted a novel expansion of “drug-excipient integration” material applications. Moreover, the SSD-based P407-SSD-Lps system functioned as a stable and efficient multifunctional liposomal delivery system, offering innovative therapeutic avenues for hepatocellular carcinoma treatment.

## Highlights

• Developed SSD-based liposomes implementing a “drug-excipient integration” strategy: SSD dually functions as structural stabilizer (cholesterol replacement) and therapeutic agent for HCC treatment.
• Engineered cholesterol-free P407-SSD-Lps using SSD, adding inherent antitumor activity of SSD.
• Optimized PA/P407-modified P407-SSD-Lps demonstrated improved stability, prolonged circulation, enhanced biocompatibility, and superior hepatocyte-specific targeting.
• Designed P407-SSD-Lps significantly enhanced HepG2 cellular uptake, inhibited proliferation, induced apoptosis, and showed potent antitumor efficacy in orthotopic HCC mouse models.
• Established as stable multifunctional delivery system, then co-delivering DOX with enhanced efficacy and maintained biocompatibility for HCC therapy in vivo.

## Graphical abstract

**Figure:**
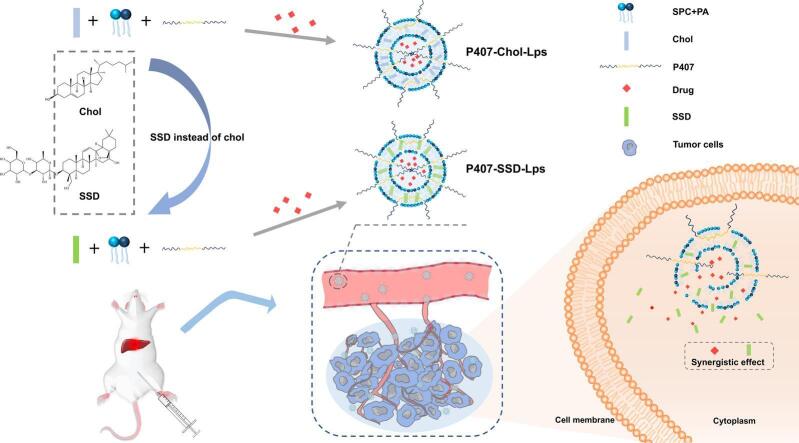

## 1 Introduction

Primary liver cancer remains a major global health challenge, with its incidence continuously rising worldwide. It currently ranks as the sixth most prevalent malignancy and the third leading cause of cancer-related mortality. Hepatocellular carcinoma, the most prevalent form of primary liver cancer, accounts for 85 %–90 % of all cases (Llovet et al., 2021; Li et al., 2024). Current clinical management of primary hepatocellular carcinoma primarily involves surgical resection, chemotherapy, and immunotherapy. However, the majority of patients lack clear surgical indications at diagnosis. Furthermore, conventional chemotherapy regimens face significant limitations due to inherent toxicity, severe adverse effects, and drug resistance (Anwanwan et al., 2020; Grabarnick Portnoy et al., 2021).

Nanoparticle-based drug delivery, a promising strategy in nanotechnology, has been extensively applied in cancer therapeutic research due to its superior tumor-targeting capacity and controlled drug release properties. This approach demonstrates potential to overcome current limitations in hepatocellular carcinoma treatment (Johnson et al., 2010; Mai et al., 2025; Chen et al., 2025; Zhang et al., 2025). Liposomes (LPs), defined as nanoscale spherical vesicles with cell membrane-mimicking bilayer structures composed of phospholipids and cholesterol, are increasingly recognized as ideal carriers for targeted anticancer drug delivery. This recognition stems from their excellent biocompatibility, capacity to reduce systemic toxicity of encapsulated drugs, and ability to enhance therapeutic outcomes (Riaz et al., 2018; Sah et al., 2025). Cholesterol (Chol), an essential component of conventional liposomes, enhances liposomal stability by modulating the fluidity of phospholipid bilayers (Ercole et al., 2015; Efimova et al., 2016; Allen and Cullis, 2013). However, accumulating evidence indicates that cholesterol may dysregulate serum lipoprotein levels and induce complement-mediated pseudoallergic reactions, potentially leading to pulmonary hypertension and other cardiopulmonary adverse effects (Moein Moghimi et al., 2008; Szebeni et al., 2000). Moreover, elevated cholesterol levels have been associated with the development of various malignancies. The degradation of conventional liposomes within tumor cells, which increases intracellular cholesterol content, may diminish antitumor efficacy or even exert counterproductive effects (Kuzu et al., 2016; Beckwitt et al., 2018; Zhang et al., 2021). Therefore, designing novel liposomal delivery systems capable of ensuring enhanced safety while improving therapeutic outcomes is imperative.

Steroidal saponins, widely distributed in traditional Chinese medicine, have attracted considerable attention due to their broad pharmacological activities, including antitumor, anti-inflammatory, and immunomodulatory effects (Dutta et al., 2019; Li et al., 2023; Emami et al., 2023). Notably, many saponins exhibit cholesterol-like structural activity that modulates the physicochemical properties of phospholipid bilayers, making them promising alternatives for cholesterol replacement in stable liposome preparation. Among these, saikosaponin D (SSD), an active component extracted from Bupleurum species, consists of a hydrophilic glycoside chain and a hydrophobic aglycone moiety. The aglycone of SSD shares a steroidal backbone structure analogous to cholesterol (Hong et al., 2020; Chen et al., 2020). SSD demonstrates remarkable antitumor efficacy through suppression of cyclooxygenase-2 (COX-2), thereby inhibiting proliferation and inducing apoptosis in diverse tumor cell types (Yang et al., 2022; Gu et al., 2025). Consequently, using SSD to replace cholesterol in liposome preparation not only replicates cholesterol's stabilizing functions but also enhances antitumor efficacy. This achieves dual functionality as both therapeutic agent and structural excipient, realizing “drug-excipient integration”.

Safety remains a critical prerequisite in pharmaceutical formulation development. Although SSD demonstrates hepatoprotective effects, prolonged high-dose administration has been reported to induce neurological, cardiac, and hemolytic toxicities (Qiu et al., 2023; Zhang et al., 2011). Poloxamer 407 (P407), an amphiphilic triblock copolymer comprising poly (ethylene oxide)-poly (propylene oxide) chains, enhances liposomal stability through its hydrophobic poly (propylene oxide) central block that anchors into lipid bilayers. Concurrently, its hydrophilic poly (ethylene oxide) terminal blocks form a hydrated surface layer, improving biocompatibility and prolonging systemic circulation (Liang et al., 2005; Wu and Lee, 2009; Minnelli et al., 2018). The enhanced targeting efficiency further reduces off-target organ toxicity. As a natural, negatively charged phospholipid, PA confers a negative surface charge to the liposomes. These negatively charged liposomes preferentially adsorb apolipoproteins and serum albumin in the bloodstream, a process which is known to facilitate preferential uptake by hepatocytes while minimizing off-target clearance (Ding et al., 2023; Nie et al., 2023; Wang et al., 2024).

**Fig. 1:**
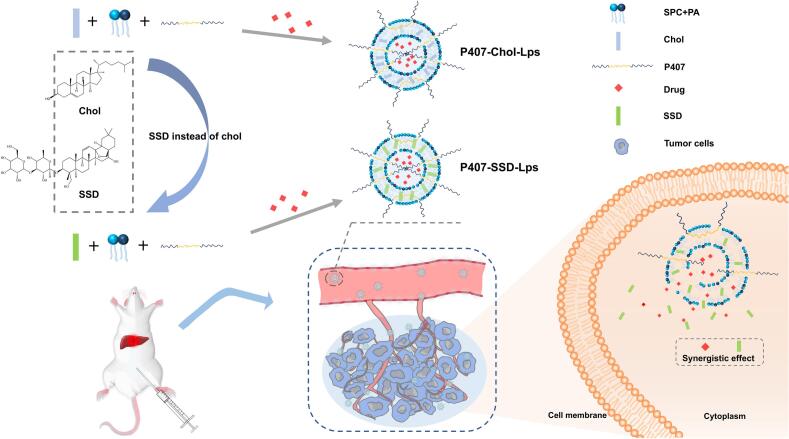
Rational design of SSD-based multifunctional liposomal delivery systems. In this system, Chol is replaced by SSD serving as both a membrane modulator and antitumor agent. Concurrent PA and P407 modification optimizes physicochemical properties and potentiates tumoricidal efficacy, enabling synergistic drug action against hepatocellular carcinoma.

In this study, we developed a novel cholesterol-free liposomal delivery system (P407-SSD-Lps) that represents a distinct approach in nanocarrier design. Unlike conventional liposomes that simply encapsulate drugs, our system is unique in that SSD serves not merely as a loaded drug but as an integral structural and functional component of the nanocarrier itself. This dual-function approach enables SSD substitution to not only circumvent the formation of a high-cholesterol microenvironment at tumor sites but also exert direct tumoricidal effects through its inherent antitumor properties within the lipid bilayer. Furthermore, by incorporating strategic modifications with PA and P407, we endowed the liposomes with enhanced stability, biocompatibility, and hepatocyte-targeting capability. This integrated drug-excipient structure, combined with rational material integration, collectively distinguishes our platform from most existing multifunctional drug delivery systems (Fig. 1).

## 2 Materials and methods

### 2.1 Materials

The SSD was purchased from Chengdu Desite Bio-Technology Co., Ltd. The DOX was obtained from Sichuan Weikeqi Biotechnology Co., Ltd. The P407 and dialysis bags were sourced from Shanghai Yeasen Biotechnology Co., Ltd. The Chol was procured from Beijing Solarbio Science & Technology Co., Ltd. The Soybean phosphatidylcholine (SPC) was supplied by Shanghai Macklin Biochemical Technology Co., Ltd. The PA was acquired from Tianjin Pusi Tang Biomedical Technology Co., Ltd. The Polysorbate 80 was provided by Shanghai Jiuxinyan Biotechnology Co., Ltd. The DiR fluorescent dye was purchased from Shanghai Absin Bioscience Inc. The Cell Counting Kit-8 (CCK-8) was obtained from Wuhan Yakeyin Biotechnology Co., Ltd. The Calcein-AM/PI Live/Dead Cell Double Staining Kit, the DMEM medium, and the trypsin were sourced from Wuhan Servicebio Technology Co., Ltd. The Fetal bovine serum (FBS), the penicillin-streptomycin, and the PBS buffer were acquired from Shanghai Datasheer Biotech Co., Ltd. The Annexin V-FITC/PI Apoptosis Detection Kit was purchased from Wuhan Pusennuo Life Technologies Co., Ltd., while all other organic solvents used were high-performance liquid chromatography (HPLC) grade, obtained from Merck KGaA (Germany).

### 2.2 Cells culture

The human hepatocellular carcinoma cell line HepG2 was provided by the Chinese Academy of Sciences cell library (Shanghai, China). The murine hepatoma cell line H22 was purchased from the China Center for Type Culture Collection (Wuhan, China). All cells were maintained in DMEM supplemented with 10 % FBS, 100 U/mL penicillin, and 100 μg/mL streptomycin, and cultured under standard conditions of 5 % CO_2_, 95 % relative humidity, and 37 °C.

### 2.3 Animals

Male Kunming mice (body weight 18–22 g) were supplied by Beijing SPF Biotechnology Co., Ltd. (Beijing, China). All experimental proceedings were conducted in compliance with the “Laboratory Animal Guideline for Ethical Review of Animal Welfare (GB/T 35892–2018)”. The protocol was approved by the Institutional Animal Ethics Committee of Yunnan University of Chinese Medicine (Approval No. YNUTCM-XMSS-G-20250051).

### 2.4 Preparation of liposomes

The cholesterol-free P407-SSD-Lps were prepared using thin-film dispersion combined with ultrasonication. Briefly, the predetermined amounts of SPC, PA, and SSD were dissolved in methanol in a round-bottom flask. The organic solvent was evaporated under reduced pressure using a rotary evaporator maintained at 40 °C to form a homogeneous lipid film. Subsequently, 5 mL of normal saline containing P407 was added for hydration over 30 min at 40 °C, yielding an opalescent liposomal suspension. The suspension was ultrasonicated (200 W, 10 min) using a probe sonicator (Ningbo Xinzhi Biotechnology Co., Ltd., China). Finally, the product was sequentially filtered through 0.45 μm and 0.22 μm microporous membranes (three cycles per filter) to obtain the final P407-SSD-Lps, whose encapsulation efficiency (*EE*)and drug loading (*DL*) were subsequently determined according to the methodology detailed in Supplementary Data.

### 2.5 Orthogonal experiment

Building on the single-factor experiments detailed in the Supplemental Data, the mass of SPC (A), the SPC/SSD ratio (B, *w*/w), and the SPC/P407 ratio (C, w/w) were identified as critical factors, each assigned three levels (Table S2). An L_9_ (3^4^) orthogonal array was employed to optimize the liposomal formulation. *EE* (*X*) and zeta potential (*Y*) served as evaluation criteria. The weighting coefficients of *X* and *Y* were determined via the entropy weight method (Cheng et al., 2021; Hao et al., 2022). The comprehensive score was calculated using Eq. (1).

(1) $$\mathrm{Comprehensive score}=\left(\frac{X\times \text{Weight coefficient}}{X_{\max}}+\frac{|Y|\times \text{Weight coefficient}}{\;{\left|Y\right|}_{\max}}\;\right)\times 100$$

### 2.6 Liposome characterization

A 10 μL aliquot of P407-SSD-Lps was applied to a 300-mesh carbon-coated copper grid and allowed to stand for 10 min. The grid was then thoroughly rinsed with distilled water and stained twice with 2 % phosphotungstic acid solution for 10 s each. After complete air-drying, the morphological characteristics of the liposomes were examined using transmission electron microscopy (TEM, Hitachi, HT7800, Japan) at an accelerating voltage of 80 kV. The particle size, polydispersity index (PDI), and zeta potential of P407-SSD-Lps and P407-Chol-Lps were measured using a nanoparticle size and zeta potential analyzer (NanoBrook 90Plus PALS, Brookhaven Instruments Corporation, USA). Furthermore, in vitro release kinetics and 30-day stability of P407-SSD-Lps were systematically investigated. Detailed experimental procedures are provided in Supplementary Data.

### 2.7 In vitro cytotoxicity and hemocompatibility

The HepG2 cells were seeded in 96-well plates at a density of 2 × 10^4^ cells/well and incubated for 24 h at 37 °C with 5 % CO_2_. The original medium was aspirated, and replaced with fresh medium containing varying concentrations of excipients, free SSD, or P407-SSD-Lps (SSD concentrations: 1.0, 2.0, 3.0, 4.0, 5.0, 6.0, 7.0, and 8.0 μg/mL). After 24 h of incubation, 10 μL of CCK-8 solution was added to each well, followed by 2 h of incubation. Absorbance (*A*) was measured at 450 nm using a microplate reader (Thermo Fisher Scientific, USA). The cell proliferation inhibition rate was calculated using Eq. (2), and the half-maximal inhibitory concentration (IC_50_) was determined with GraphPad Prism 9.5 software.

Furthermore, the hemocompatibility of P407-SSD-Lps at concentrations of 1.0, 2.0, 3.0, 5.0, and 8.0 μg/mL was evaluated. Detailed experimental procedures are provided in the Supplementary Data.

(2) $$\mathrm{Cell proliferation inhibition rate}\;\left(\%\right)=\frac{\left(A\text{control}–A\text{test}\right)}{\left(A\text{control}–A\text{blank}\right)}\times 100\%$$

### 2.8 Live/dead cell staining experiment

The free SSD and P407-SSD-Lps were diluted with cell culture medium to final SSD concentrations of 1.0, 3.0, and 5.0 μg/mL. HepG2 cells were seeded in 96-well plates at 5 × 10^4^ cells/well and incubated for 24 h. The original medium was aspirated and replaced with fresh medium containing the drug solutions, followed by another 24 h of incubation. Diluted Calcein-AM/PI solution was added to each well and incubated in the dark for 20 min. Finally, the staining solution was removed, and cells were imaged under an inverted fluorescence microscope (Leica Microsystems GmbH, Germany).

### 2.9 Scratch wound healing assay

To evaluate the effects of free SSD and P407-SSD-Lps on the HepG2 cells migration, a scratch assay was performed. HepG2 cells were digested, counted, and diluted to 1 × 10^6^ cells/mL, with 1 mL aliquots seeded into 12-well plates. When cells reached 80–90 % confluence, the supernatant was removed, and a straight scratch was created using a 10 μL pipette tip. Residual cells were gently washed away with PBS. Subsequently, DMEM medium containing free SSD or P407-SSD-Lps was added for incubation. After 24 h and 48 h, residual cells were washed, and cell migration was imaged using an inverted fluorescence microscope. Scratch areas were analyzed with Image J software, and the wound healing rate was calculated using Eq. (3).

(3) $$\mathrm{Wound healing rate}\;\left(\%\right)=\frac{\left(A_{0}–A_{t}\right)}{A_{0}}\times 100\%$$

where *A*_0_ represents the scratch area at 0 h, and *A*_t_ denotes the scratch area at 24 h or 48 h.

### 2.10 In vitro cell apoptosis

The HepG2 cells were seeded in 6-well plates at a density of 1 × 10^6^ cells/well and incubated for 24 h. The medium was aspirated and replaced with fresh medium containing free SSD or P407-SSD-Lps, followed by another 24 h of incubation. Cells were washed with PBS, centrifuged at 1200 rpm for 5 min, and the pellet was resuspended in 500 μL of 1× Binding Buffer. Subsequently, 5 μL of Annexin V-FITC and 5 μL of PI staining solution were added, mixed gently, and incubated in the dark for 10 min. Apoptosis analysis was performed using a flow cytometer (Agilent Technologies, Inc., USA).

### 2.11 Animal models

The H22 cells were diluted to 3 × 10^7^ cells/mL with sterile PBS. A 0.2 mL aliquot of the cell suspension was intraperitoneally injected into mice for in vivo expansion. Upon observing significant abdominal distension, ascitic fluid was collected under aseptic conditions and centrifuged at 1500 rpm for 4 min. The supernatant was discarded, and the H22 ascites cells were washed with sterile PBS. The cell density was adjusted to 1 × 10^7^ cells/mL, and the suspension was inoculated into the mouse liver using a microsyringe to establish a murine orthotopic hepatocellular carcinoma transplantation model (Cheng et al., 2024).

### 2.12 In vivo targeting

Tumor-bearing mice were randomly divided into two groups (*n* = 6) to receive either P407-DiR-Chol-Lps or P407-DiR-SSD-Lps, respectively. After intravenous administration (0.01 mL/g dose), mice were anesthetized with isoflurane. Fluorescence imaging of the abdominal region was performed at predefined time points (1, 2, 4, 8, 12, 24, 48, and 72 h) using an in vivo imaging system (IVIS, PerkinElmer, Inc., USA) under identical acquisition settings. At 72 h post-injection, mice were humanely euthanized, and tumors along with heart, liver, spleen, lungs, kidneys, and brain were excised, rinsed with saline, and subjected to ex vivo fluorescence imaging. Optimal signal detection was achieved using excitation/emission wavelengths of 750 nm and 782 nm, respectively. Additionally, quantitative analysis of fluorescence images was conducted using the IVIS imaging system (Liu et al., 2022; Liu et al., 2019). The tumor targeting index (TTI) at each time point during in vivo imaging was calculated using Eq. (4). Finally, the area under the TTI-time curve (AUTC) was determined via the trapezoidal rule, and the fluorescence intensity ratios of tumors to other organs were computed using Eq. (5).

(4) $$\mathrm{TTI}\;\left(\%\right)=\frac{\text{Fluorescence intensity of tumor region}}{\text{Fluorescence intensity of whole body}}\times 100\%$$

(5) $$\mathrm{Intensity ratio}=\frac{\text{Fluorescence intensity of tumor}}{\text{Fluorescence intensity of organ}}$$

### 2.13 In vivo antitumor efficacy and biocompatibility

Initially, the P407-SSD-Lps and DOX-loaded liposomes (P407-DOX-SSD-Lps) were prepared using the optimized prescription and process to comparatively evaluate the therapeutic efficacy of free drugs versus both liposomal systems in tumor-bearing mice. Successful model establishment was confirmed on day 3 post-inoculation via hepatic ultrasonography and macroscopic examination of excised livers (Cheng et al., 2024). Tumor-bearing mice were randomly divided into six groups (*n* = 7 per group): blank group, model group, free DOX group, free SSD group, P407-SSD-Lps group, and P407-DOX-SSD-Lps group, respective. The treatments were initiated on day 3: the blank and model groups received normal saline (NS), while the remaining groups were administered free DOX, free SSD, P407-SSD-Lps, or P407-DOX-SSD-Lps (SSD dose: 10 mg/kg; DOX dose: 2 mg/kg) (Luo et al., 2024; Zhang et al., 2019; Jiang et al., 2022). Treatments were administered intraperitoneally every other day for a total of seven doses. Tumor suppression in each group was monitored via B-mode ultrasonography (Shenzhen Mindray Bio-Medical Electronics Co., Ltd., China) on days 5, 11, and 16 post-treatment initiation. Then, mice were anesthetized with isoflurane for blood collection. Complete blood count (CBC) analysis was performed using an automated hematology analyzer (Shenzhen Mindray Bio-Medical Electronics Co., Ltd., China). Serum levels of AFP, GPC-3, DCP, CEA, TNF-α, IL-6, IL-1β, ALT, AST, Crea, BUN, CK, and LDH were quantified using ELISA kits (Jiangsu Meimian Industrial Co., Ltd., China) and an automated biochemical analyzer (Shandong Biobase Biotechnology Co., Ltd., China). Following humane euthanasia, mice were dissected to evaluate abdominal adhesions, ascites presence, and abdominal wall tumors. Excised livers were photographed. The tumor volume and tumor weight were recorded. Tumor growth inhibition rates based on weight (TGI_w_) and volume (TGI_v_) were calculated using Eqs. (6), (7), respectively. Statistically, TGI < 40 % indicated no antitumor efficacy, while TGI ≥ 40 % with *p* < 0.05 denoted significant tumor suppression. Finally, organs and tissues were fixed in 4 % paraformaldehyde, paraffin-embedded, sectioned, and stained with hematoxylin and eosin (H&E) following standard protocols.

(6) $${\mathrm{TGI}}_{w}=\frac{W_{\text{model}}–W_{\text{drug}}}{W_{\text{model}}}\times 100\%$$

(7) $${\mathrm{TGI}}_{v}=\frac{V_{\text{model}}–V_{\text{drug}}}{V_{\text{model}}}\times 100\%$$

### 2.14 Statistical analysis

Experimental data are expressed as mean ± standard deviation (SD) and were assessed using Student's *t*-test. One-way analysis of variance (ANOVA) was performed with SPSS (22.0) software. Statistical significance was defined as p < 0.05 in all analyses.

## 3 Results

### 3.1 Orthogonal experiment

The entropy weight method assigned weighting coefficients of 0.505 8 and 0.494 2 to *EE* (*X*) and zeta potential (*Y*), respectively. Detailed results of the L_9_ (3^4^) orthogonal array design and analysis of variance are presented in Table 1, Table 2. The influence of factors on the comprehensive score followed the order C > A > B, with factors A and C exhibiting statistically significant effects (p < 0.05). The optimal formulation was determined as A_1_B_1_C_1_ with SPC mass = 25 mg, SPC/SSD = 11:1, and SPC/P407 = 2:1. Validation experiments under these optimized conditions (Table 3) yielded a mean comprehensive score of (97.23 ± 1.20), confirming the robustness and reproducibility of the preparation process for P407-SSD-Lps.

**Table 1:** The L_9_ (3^4^) orthogonal test table and results.

	A	B	C	D (Error)	X (%)	Y (mV)	Comprehensive score
1	1	1	1	1	80.41	−53.35	99.26
2	1	2	2	2	81.60	−50.56	97.42
3	1	3	3	3	77.69	−45.88	90.66
4	2	1	2	3	81.37	−47.73	94.66
5	2	2	3	1	79.38	−42.61	88.68
6	2	3	1	2	79.73	−47.05	93.00
7	3	1	3	2	77.55	−43.84	88.68
8	3	2	1	3	79.79	−50.26	96.02
9	3	3	2	1	81.05	−46.36	93.19
K1	95.78	94.20	96.10	93.71			
K2	92.11	94.04	95.09	93.04			
K3	92.63	92.28	89.34	93.78			
R	3.67	1.92	6.75	6.75

**Table 2:** Results of variance analysis.

Source	Sum of squares	Degrees of freedom	Mean square	F-value	*P*-value
A	23.654	2	11.827	23.284	0.041
B	6.782	2	3.391	6.676	0.130
C	79.657	2	39.828	78.411	0.013
D (Error)	1.016	2	0.508

**Table 3:** Results of validation experiments.

Number	EE (%)	DL (%)	Zeta potential (mV)	Particle size (nm)	PDI	Comprehensive Score
1	81.84	3.31	−50.34	127.27	0.186	97.37
2	80.52	4.18	−52.42	130.14	0.184	98.47
3	78.36	3.69	−51.27	126.25	0.146	96.07
Mean	80.24 ± 1.76	3.54 ± 0.42	−51.34 ± 1.04	127.89 ± 2.02	0.172 ± 0.023	97.23 ± 1.20

### 3.2 Characterization of liposomes

**Fig. 2:**
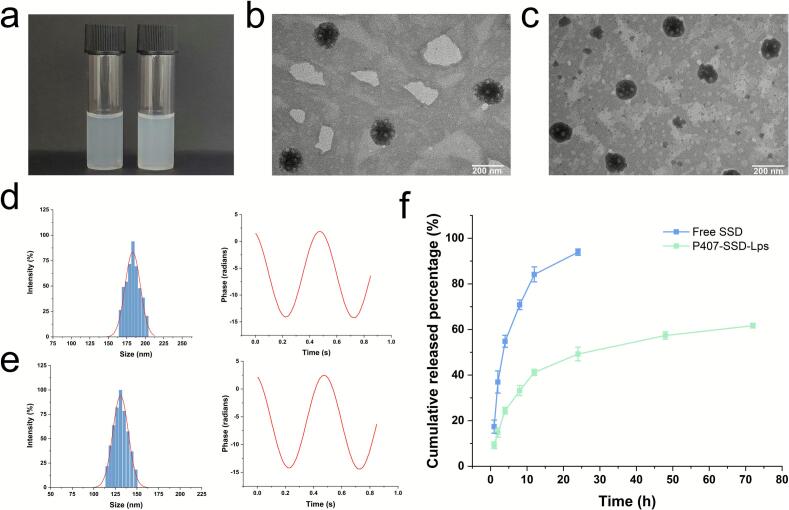
Characterization of liposomes. (**a**) Macroscopic appearance of P407-Chol-Lps (left) and P407-SSD-Lps (right). (**b)** Morphological characteristics of P407-Chol-Lps (scale bar = 200 nm). (**c**) Morphological characteristics of P407-SSD-Lps (scale bar = 200 nm). (**d**) Particle Size Distribution and Zeta Potential of P407-Chol-Lps. (**e**) Particle Size Distribution and Zeta Potential of P407-SSD-Lps. (**f**) In vitro release curve (mean ± SD, *n* = 3).

The liposomes prepared via the optimized protocol appeared as homogeneous opalescent liquids (Fig. 2a). TEM imaging revealed that both P407-SSD-Lps and P407-Chol-Lps maintained well-defined spherical morphology with uniform dispersion (Fig. 2b and c). Both liposomal formulations exhibited narrow, monomodal size distributions. P407-SSD-Lps showed a mean particle size of (127.89 ± 2.02) nm, which was significantly smaller than that of P407-Chol-Lps (185.49 ± 8.90) nm (p < 0.001), with respective PDIs of 0.172 ± 0.023 (P407-SSD-Lps) and 0.260 ± 0.024 (P407-Chol-Lps). The zeta potentials were (−51.34 ± 1.04) mV for P407-SSD-Lps and (−54.70 ± 3.04) mV for P407-Chol-Lps, indicating negatively charged surfaces and stable colloidal dispersions (Fig. 2d and e). The EE and DL capacity of SSD in P407-SSD-Lps were (80.24 ± 1.76)% and (3.54 ± 0.42)%, respectively (Table 3).

Furthermore, free SSD exhibited rapid release kinetics, achieving a cumulative release rate of (93.89 ± 1.42)% within 24 h. In contrast, P407-SSD-Lps demonstrated sustained and controlled release, with a cumulative release of (61.66 ± 1.04)% over 72 h (Fig. 2f). This result indicated that liposomes prepared by substituting cholesterol with SSD in combination with phospholipids can exert moderate sustained-release effects. Fitting of release kinetics revealed that the drug release mechanism of P407-SSD-Lps conformed to the Weibull model (Table S3).

P407-SSD-Lps showed no visible changes in appearance during storage. Minor variations in *EE*, particle size, PDI, and zeta potential were observed over 30 days, with PDI consistently <0.3 and zeta potential absolute values maintained above 40 mV (Table S4). These findings indicated that no aggregation occurred between liposomal particles during storage, demonstrating high storage stability.

### 3.3 In vitro anti-tumor activity and hemocompatibility

**Fig. 3:**
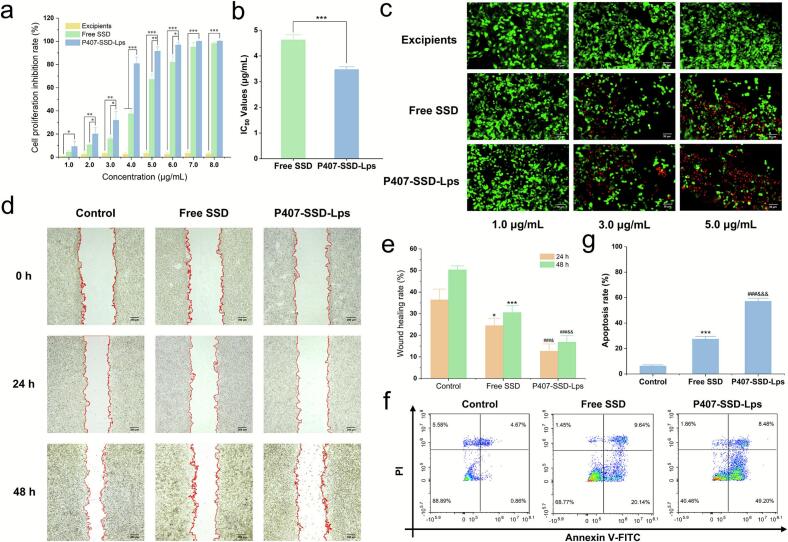
In vitro antitumor efficacy evaluation of P407-SSD-Lps. (**a**) Proliferation inhibitory effects of Excipients, Free SSD, and P407-SSD-Lps on HepG2 Cells (mean ± SD, *n* = 3). (**b**) IC_50_ Values of Free SSD and P407-SSD-Lps (mean ± SD, n = 3). (**c**) Live/Dead Cell Staining of Excipients, Free SSD, and P407-SSD-Lps. The images were obtained at 200× magnification (scale bar = 50 μm). Green fluorescence indicates viable cells, while red fluorescence indicates dead cells. (**d**) Effects of Free SSD and P407-SSD-Lps on HepG2 cells migration. The images were obtained at 40× magnification (scale bar = 200 μm). (**e**) Scratch healing rates of HepG2 cells at 24 h and 48 h (mean ± SD, n = 3). (**f**) Apoptosis induction in HepG2 cells by Free SSD and P407-SSD-Lps. (**g**) Apoptosis rates of HepG2 cells (mean ± SD, n = 3). (For interpretation of the references to colour in this figure legend, the reader is referred to the web version of this article.) **Note:** In figures (**a**, **b**), ^⁎^*p* < 0.05, ^⁎⁎^ p < 0.01, ^⁎⁎⁎^*p* < 0.001. In figure (**e**), Free SSD group. vs Control group, ^⁎^ p < 0.05, ^⁎⁎⁎^ p < 0.001. P407-SSD-Lps group. vs Control group, ^##^ p < 0.01, ^###^ p < 0.001. P407-SSD-Lps group. vs Free SSD group, ^&^ p < 0.05, ^&&^*p* < 0.01. In figure (**g**), Free SSD group. vs Control group, ^⁎⁎⁎^ p < 0.001. P407-SSD-Lps group. vs Control group, ^###^ p < 0.001. P407-SSD-Lps group. vs Free SSD group, ^&&&^ p < 0.001.

The in vitro antitumor activity of liposomal excipients, free SSD, and P407-SSD-Lps was evaluated using the CCK-8 assay. As shown in Fig. 3a, both free SSD and P407-SSD-Lps exhibited significant concentration-dependent proliferation inhibitory effects on HepG2 cells, whereas the excipients showed no such activity. At SSD concentrations of 2.0–6.0 μg/mL, P407-SSD-Lps demonstrated stronger antiproliferative effects compared to free SSD after 24 h of treatment (*p* < 0.05). The IC_50_ values of free SSD and P407-SSD-Lps against HepG2 cells were calculated as (4.626 ± 0.197) μg/mL and (3.469 ± 0.104) μg/mL, respectively. Compared to free SSD, P407-SSD-Lps exhibited a 1.33-fold reduction in IC_50_ (Fig. 3b). Additionally, live/dead cell staining (Fig. 3c) was employed to assess cytotoxicity. The excipients showed negligible toxicity toward HepG2 cells within 24 h, while both free SSD and P407-SSD-Lps demonstrated concentration-dependent inhibitory effects. Notably, P407-SSD-Lps induced the highest cytotoxicity.

Given the potent hemolytic toxicity of SSD (Francis et al., 2002), we further evaluated the hemocompatibility of P407-SSD-Lps. As shown in Fig. S3, free SSD induced >5 % hemolysis at 2 μg/mL, indicating significant erythrocyte damage. In contrast, P407-SSD-Lps consistently maintained hemolytic rates <5 % across the tested concentration range (1–8 μg/mL), demonstrating superior blood compatibility. This phenomenon was likely attributed to P407-mediated stabilization of the liposomal bilayer structure, which effectively reduced drug leakage, combined with the formation of a dense hydration barrier on the liposomal surface that prevented direct SSD-erythrocyte contact (Liang et al., 2005). Collectively, these results confirm that P407-modified SSD-based liposomes significantly enhance in vitro antitumor activity while preserving excellent hemocompatibility.

### 3.4 Scratch wound healing assay

The effects of different treatment groups on HepG2 cells migration are shown in Fig. 3d and e. Wound healing assay results revealed that untreated control cells exhibited substantial wound closure, with a healing rate of (50.28 ± 1.85)% at 48 h. In contrast, the cells treated with free SSD and P407-SSD-Lps showed significantly reduced healing rates of (30.50 ± 3.23)% and (16.80 ± 3.05)% (*p* < 0.001), respectively. These findings indicated that both formulations inhibit HepG2 cells migration, and the inhibitory effect was significantly stronger when SSD was prepared in liposomal form compared to free SSD (*p* < 0.01).

### 3.5 In vitro cell apoptosis

Apoptosis assay results are shown in Fig. 3f and g. HepG2 cells treated with free SSD or P407-SSD-Lps at equivalent concentrations for 24 h exhibited varying degrees of apoptosis. The apoptosis rate in the free SSD group was (27.39 ± 2.10)%, whereas the P407-SSD-Lps group showed a significantly increased apoptosis rate of (57.15 ± 2.41)% (*p* < 0.05), demonstrating enhanced pro-apoptotic efficacy of P407-SSD-Lps. These findings aligned with the trends observed in cytotoxicity and live/dead cell staining assays, confirming that liposomal formulation of SSD potentiated its tumor cell-killing effects.

### 3.6 Tumor targeting study

**Fig. 4:**
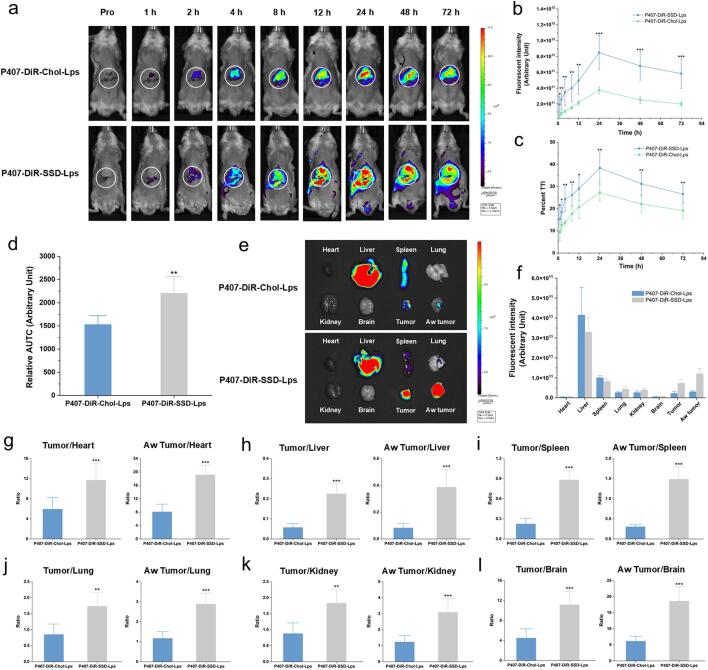
In vivo targeting study. (**a**) Fluorescence imaging post-intravenous administration of liposomal formulations in tumor-bearing mice. The white circle indicates the tumor site. (**b**) Tumor-region fluorescence intensity profiles across groups (mean ± SD, *n* = 6). (**c**) TTI curves of different groups (mean ± SD, n = 6). (**d**) AUTC values of different groups (mean ± SD, n = 6). (**e**) Ex vivo fluorescence imaging of tumors, abdominal wall tumors, and organs/tissues across groups. (**f**) Quantitative analysis of ex vivo fluorescence images (mean ± SD, n = 6). (**g**-**l**) Fluorescence intensity ratios of tumors/abdominal wall tumors to Heart (**g**), Liver (**h**), Spleen (**i**), Lung (**j**), Kidney (**k**), Brain (**l**) (mean ± SD, n = 6). **Note:** vs P407-DiR-Chol-Lps group, ^⁎^*p* < 0.05, ^⁎⁎^*p* < 0.01, ^⁎⁎⁎^*p* < 0.001.

The targeting capability of P407-DiR-SSD-Lps toward HepG2 cells was first assessed through in vitro cellular uptake studies. As shown in Fig. S4, the P407-DiR-SSD-Lps group exhibited stronger fluorescence intensity than the P407-DiR-Chol-Lps group at both 6 h and 12 h post-incubation. Therefore, the biodistribution and tumor-targeting capabilities of both P407-DiR-Chol-Lps and P407-DiR-SSD-Lps were further evaluated in tumor-bearing mice using an IVIS imaging system. As shown in Fig. 4a, both liposomal systems generated detectable fluorescence signals in tumor regions following intravenous administration. In the P407-DiR-Chol-Lps group, fluorescence intensity in tumor regions gradually increased, peaked at 24 h, and subsequently declined. Conversely, the P407-DiR-SSD-Lps group exhibited stronger fluorescence signals starting from 1 h, which plateaued at 24 h and remained strongly detectable at 72 h. These results demonstrated that P407-DiR-SSD-Lps achieved superior tumor targeting and prolonged intratumoral accumulation.

To more accurately evaluate the targeting capability of P407-DiR-SSD-Lps, the fluorescence images were quantitatively analyzed using Living Image (4.7.2) software. The tumor fluorescence intensity curves (Fig. 4b) revealed that P407-DiR-SSD-Lps maintained higher fluorescence values in tumor regions compared to P407-DiR-Chol-Lps over 72 h, consistent with the trends observed in Fig. 4a. Statistically significant differences between the two groups were observed at all time points (*p* < 0.05). Since elevated fluorescence intensity values alone could not directly demonstrate effective tumor targeting by liposomes, the TTI percentage values enabled a more precise assessment of whether tumor-region fluorescence genuinely reflected drug accumulation within tumors. Therefore, we further assessed the targeting capability of the P407-DiR-SSD-Lps by calculating TTI percentage values. As shown in Fig. 4c, the mean TTI percentage values of the P407-DiR-SSD-Lps group exceeded those of the P407-DiR-Chol-Lps group at all observed time points, confirming superior tumor-targeting efficacy. The results were consistent with the trends in Fig. 4b and displayed statistically significant intergroup differences (p < 0.05). Furthermore, to facilitate comparative analysis, the area under the TTI-time curve (AUTC) was adopted as a comprehensive metric to evaluate the overall tumor-targeting efficacy of the liposomes, thereby addressing the limitation that TTI percentage values only reflect targeting capacity at discrete time points. As shown in Fig. 4d, the AUTC of the P407-DiR-SSD-Lps group was significantly greater than that of the P407-DiR-Chol-Lps group (p < 0.05). These demonstrated that P407-DiR-SSD-Lps enhanced tumor-targeting capability, promoted drug accumulation at lesion sites, and thereby potentiated therapeutic efficacy against tumors.

It should be noted that background interference from fur and non-target tissues during in vivo imaging may compromise fluorescence signal accuracy. To enable precise comparison of the two liposomal systems, mice were humanely euthanized at 72 h post-intravenous injection, and ex vivo organs or tissues were imaged under identical acquisition settings. As shown in Fig. 4e, the P407-DiR-Chol-Lps group exhibited strong fluorescence signals in the liver and spleen but minimal tumor-associated fluorescence, indicating that in vivo signals predominantly originated from hepatic and splenic retention. In contrast, although not discernible during the in vivo imaging, ex vivo organ analysis revealed that the P407-DiR-SSD-Lps group exhibited strong fluorescence signals in the liver and spleen, in addition to intense tumor fluorescence. Furthermore, quantitative analysis was performed on each tumor and organ or tissue. The fluorescence intensity of tumors in the P407-DiR-SSD-Lps group exceeded that of the P407-DiR-Chol-Lps group, consistent with in vivo imaging observations. Notably, tumors in the P407-DiR-SSD-Lps group exhibited significantly higher fluorescence intensities compared to non-hepatic/splenic organs, whereas tumors in the P407-DiR-Chol-Lps group showed fluorescence levels similar to non-hepatic/splenic organs (Fig. 4f). These indicated that P407-DiR-SSD-Lps preferentially accumulated in tumors with reduced off-target deposition in normal organs or tissues. Additionally, the fluorescence intensity ratios of hepatic tumor or abdominal wall tumor (Aw tumor) to heart, liver, spleen, lungs, kidneys, and brain were significantly higher in the P407-DiR-SSD-Lps group than in the P407-DiR-Chol-Lps group (Fig. 4g-4l). These results demonstrated that P407-DiR-SSD-Lps achieved superior in vivo tumor-targeting efficacy. This phenomenon might be attributed to SSD's ability to promote cellular autophagy and mitochondrial function, which subsequently upregulated organic anion transporting polypeptide 1B1 expression and reduced tumor extracellular matrix deposition, thereby enhancing drug distribution within tumor regions (Xu et al., 2021; Chen et al., 2023).

### 3.7 Antitumor efficacy

**Fig. 5:**
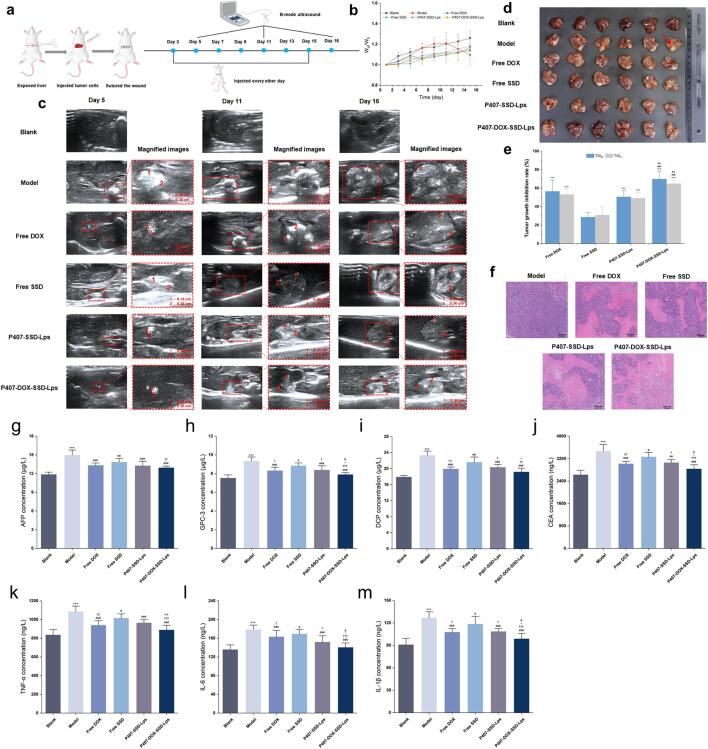
In vivo therapeutic efficacy assessment. (**a**) Schematic illustration of treatment regimens. (**b**) Body weight changes across groups (mean ± SD, *n* = 7). (**c**) B-ultrasound imaging of experimental groups. Images on the right at each time point correspond to magnified views of the tumors (**d**) Tumor status in mice post-treatment. The red arrow shows the tumor site. (**e**) Tumor inhibition rates of treatment groups (mean ± SD, n = 7). (**f**) H&E-stained sections of tumors from different treatment groups (scale bar = 100 μm). (**g**) AFP. (**h**) GPC-3. (**i**) DCP. (**j**) CEA. (**k**) TNF-α. (**l**) IL-6. (**m**) IL-1β. (For interpretation of the references to colour in this figure legend, the reader is referred to the web version of this article.) **Note:** In figure (**e**), vs Free SSD group, ^⁎⁎⁎^*p* < 0.001. vs P407-SSD-Lps group, ^###^ p < 0.001. vs Free DOX group, ^&&^*p* < 0.01. In figures (**g**-**m**), Model group. vs Blank group, ^⁎⁎⁎^ p < 0.001. vs Model group, ^#^*p* < 0.05, ^##^ p < 0.01, ^###^ p < 0.001. vs Free SSD group, ^†^p < 0.05, ^††^p < 0.01, ^†††^p < 0.001. vs P407-SSD-Lps group, ^^^ p < 0.05, ^^^^*p* < 0.01. vs Free DOX group, ^&^*p* < 0.05. Data are presented as mean ± SD (n = 7).

The P407-DOX-SSD-Lps prepared using the optimized prescription exhibited a particle size of (133.73 ± 3.91) nm, a zeta potential of (−48.92 ± 2.02) mV, and a PDI of (0.220 ± 0.020). The *EE* of SSD and DOX were (80.49 ± 1.68)% and (78.09 ± 1.90)%, respectively, while the *DL* capacities were (3.76 ± 0.18)% and (2.48 ± 0.19)%, respectively. These results indicated that the liposomes did not undergo significant changes after the loading of DOX. The in vivo antitumor efficacy of P407-SSD-Lps in murine orthotopic hepatocellular carcinoma transplantation models was evaluated (Fig. 5a). Body weight was monitored every other day post-modeling. To account for inter-individual variability in initial weight, changes were expressed as the ratio of W_d_ to W_0_, where W_d_ represents the body weight at specific time points, and W_0_ denotes the initial body weight. As shown in Fig. 5b, the model group exhibited progressive weight gain until peaking at day 11, followed by decline accompanied by lethargy, reduced food intake, and poor mental status. Conversely, the blank control group maintained steady weight gain, and all treatment groups showed no significant weight loss with preserved physiological activity throughout the study, confirming favorable biocompatibility of the liposomal systems.

B-ultrasound imaging, a widely used screening modality for hepatocellular carcinoma, offers advantages of real-time capability, safety, and cost-effectiveness compared to other imaging techniques (Singal et al., 2023). To monitor tumor progression, serial B-ultrasound scans were performed during the treatment period. Imaging revealed homogeneous medium echogenicity with clear intrahepatic ductal structures and absence of focal lesions in the blank control group. Other groups exhibited mixed-echo mass lesions with ill-defined margins, suggesting infiltrative tumor growth. Progressive tumor enlargement was observed in all groups over time. However, all treatment groups showed attenuated tumor growth compared to the model group, with the P407-DOX-SSD-Lps group demonstrating the most significant suppression (Fig. 5c). These indicated that P407-DOX-SSD-Lps exerted notable inhibitory effects on tumor progression.

Gross examination revealed severe pathological manifestations in the model group, including abdominal wall tumors, extensive peritoneal adhesions, and copious bloody ascites. In contrast, all treatment groups exhibited significant reductions in the incidence of abdominal wall tumors, ascites formation, and severity of peritoneal adhesions, with the P407-DOX-SSD-Lps group demonstrating the most pronounced therapeutic effects (Tables S5 and S6). Additionally, post-euthanasia livers were excised for photographic documentation, tumor volume measurement, and weighing. As anticipated, P407-DOX-SSD-Lps treatment demonstrated superior tumor suppression compared to Free SSD, P407-SSD-Lps, and Free DOX (Fig. 5d). Analysis revealed that P407-DOX-SSD-Lps achieved higher tumor growth inhibition rates than other groups, with TGI_V_ at (65.50 ± 2.58)% and TGI_W_ at (69.87 ± 8.43)%. Specifically, volume-based TGI_V_ values were 2.44-fold, 1.40-fold, and 1.24-fold higher than those of Free SSD, P407-SSD-Lps, and Free DOX groups, respectively. Weight-based TGI_W_ values were 2.12-fold, 1.33-fold, and 1.23-fold higher than the corresponding groups (Fig. 5e). Histopathological analysis of H&*E*-stained tumor tissues revealed that tumor cells in the model group exhibited intact morphology with well-defined borders, densely packed arrangement, and sheet-like distribution without observable necrosis. In contrast, all treatment groups showed significant pathological alterations including cellular membrane rupture, karyolysis, and nuclear pyknosis, accompanied by disorganized architecture and irregular cellular morphology. Notably, the P407-DOX-SSD-Lps group demonstrated extensive tumor necrosis, indicating superior antitumor efficacy compared to other treatment groups (Fig. 5f). These results indicated that P407-DOX-SSD-Lps significantly enhanced antitumor efficacy through synergistic interactions of DOX and SSD.

The in vivo antitumor effects were further assessed by measuring serum levels of AFP, GPC-3, DCP, CEA, TNF-α, IL-6, and IL-1β via ELISA. Combined detection of serum tumor biomarkers could enhance diagnostic efficacy and provide critical guidance for evaluating tumor progression, metastatic risk, and therapeutic response (Xu, 2022). The model group exhibited significantly elevated AFP, GPC-3, DCP, and CEA levels compared to the blank control group (*p* < 0.001). All treatment groups showed reduced biomarker levels relative to the model group, with the most pronounced decreases observed in the P407-DOX-SSD-Lps group (p < 0.001, Figs. 5g-5j). Moreover, dysregulated expression of TNF-α, IL-6, and IL-1β was closely associated with altered cellular microenvironments and tumor pathological progression (Wu, 2019). ELISA results demonstrated that TNF-α, IL-6, and IL-1β levels in the model group were significantly elevated versus the blank control group (p < 0.001). All treatment groups exhibited reduced cytokine levels compared to the model group, with the P407-DOX-SSD-Lps group showing the most pronounced reduction (p < 0.001, Figs. 5k-5m). These findings demonstrated that P407-DOX-SSD-Lps ameliorated the tumor microenvironment, attenuated inflammatory responses, significantly inhibited tumor growth and metastasis, and exhibited favorable biocompatibility.

### 3.8 Biocompatibility

**Fig. 6:**
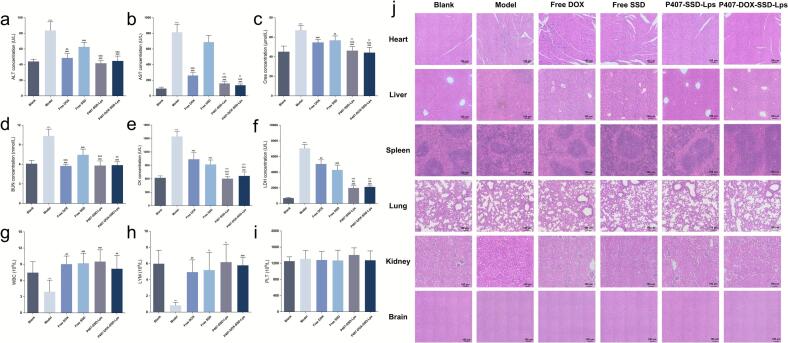
In vivo biocompatibility assessment. (**a**-**f**) Serum biomarker levels for hepatic, renal, and cardiac functions across groups. (**g**-**i**) Complete blood count analyses. (**j**) Histological examination of organs/tissues with H&E staining (scale bar = 100 μm). **Note:** (**a**) ALT. (**b**) AST. (**c**) Crea. (**d**) BUN. (**e**) CK. (**f**) LDH. (**g**) WBC. (**h**) LYM. (**i**) PLT. In figures (**a**-**i**), Model group. vs Blank group, ^⁎⁎^*p* < 0.01, ^⁎⁎⁎^*p* < 0.001. vs Model group, ^#^ p < 0.05, ^##^ p < 0.01, ^###^ p < 0.001. vs Free SSD group, ^&&^ p < 0.01, ^&&&^ p < 0.001. vs Free DOX group, ^††^p < 0.01, ^†††^p < 0.001. Data are presented as mean ± SD (*n* = 6).

Excellent biocompatibility constitutes a fundamental requirement for clinical translation of novel delivery systems (Gao et al., 2025). In this study, we evaluated the biocompatibility of liposomes by assessing hepatic, renal, and cardiac function markers alongside complete blood counts. Compared to the blank control group, the model group exhibited significantly elevated levels of ALT, AST, Crea, BUN, CK, and LDH (p < 0.001), indicating hepatic, renal, and cardiac impairment. This might be attributed to direct tumor invasion/metastasis, metabolic dysregulation, and systemic inflammatory responses, which were clinical manifestations consistent with malignant ascites (Jiang et al., 2018). Compared to the model group, Free DOX and Free SSD groups showed reductions in ALT, AST, Crea, BUN, CK, and LDH levels. However, these effects were less pronounced than those observed with P407-SSD-Lps and P407-DOX-SSD-Lps (Figs. 6a-6f). This indicated that P407-SSD-Lps and P407-DOX-SSD-Lps exerted no significant toxicity on hepatic, renal, or cardiac function while effectively mitigating organ damage in tumor-bearing mice. Complete blood count analysis revealed no intergroup differences in platelet (PLT) counts (*P* > 0.05). The model group exhibited significantly decreased white blood cell (WBC) and lymphocyte (LYM) counts versus the normal group (*p* < 0.05), whereas all treatment groups maintained WBC and LYM levels comparable to the normal group (*p* > 0.05). These findings supported the excellent biocompatibility profiles of P407-SSD-Lps and P407-DOX-SSD-Lps (Figs. 6g-6i).

Furthermore, H&E staining of major organs from tumor-bearing mice was performed post-treatment to comprehensively evaluate liposomal biocompatibility. As shown in Fig. 6j, the model group exhibited multi-organ damage. In cardiac tissue, this manifested as inflammatory cell infiltration, cardiomyocyte necrosis, and structural disarray. In hepatic tissue, this manifested as invasive tumor growth with densely packed cells, enlarged hyperchromatic nuclei, increased nuclear-to-cytoplasmic ratios, and inflammatory infiltration. In renal tissue, this manifested as necrotic damage featuring extensive inflammatory infiltration, glomerular basement membrane thickening, tubular atrophy, and luminal stenosis. In contrast, P407-SSD-Lps and P407-DOX-SSD-Lps groups demonstrated significantly ameliorated pathological architectures in heart, liver, and kidneys with markedly reduced inflammatory and tumor cells, while spleen, lungs, and brain displayed no observable pathological alterations indistinguishable from the normal group. These results confirmed the favorable biocompatibility of both liposomal formulations.

## 4 Discussion

We successfully developed a novel SSD-based liposomal delivery system embodying an innovative “drug-excipient integration” strategy. Unlike conventional liposomes relying on cholesterol for membrane stability, our system utilizes SSD both as a structural component of liposomes and as an active therapeutic agent. Through systematic optimization via single-factor and orthogonal experiments, liposomes with desirable physicochemical properties were prepared. TEM imaging revealed well-defined spherical morphology, uniform dispersion, sustained release characteristics, and high storage stability, further confirming the feasibility of this preparation strategy. In vitro and in vivo experiments demonstrated the superior antitumor efficacy of P407-SSD-Lps, attributable to several advantageous properties. Primarily, significantly enhanced cellular uptake, proliferation inhibition, and potent pro-apoptotic effects observed in HepG2 cells indicated that the liposomal formulation of the SSD not only stabilized the liposomal structure but also exerted potent antitumor activity in vitro. In an orthotopic liver cancer model, P407-DiR-SSD-Lps exhibited superior tumor targeting and prolonged intratumoral accumulation compared to P407-DiR-Chol-Lps, indicating that SSD, as a structural component of liposomes, plays a critical role in tumor targeting during cancer therapy. Furthermore, modification of liposomes with P407 and PA further enhanced the applicability of this delivery system by prolonging circulation time, improving structural stability, ensuring safety, and enhancing tumor penetration and retention. Notably, comprehensive analyses including ultrasound monitoring, tumor histopathology, serum tumor markers, and inflammatory cytokine levels revealed that the P407-DOX-SSD-Lps group achieved a significantly higher tumor growth inhibition rate compared to other treatment groups, with serum tumor markers and inflammatory cytokine levels approaching those of the blank control group. Histopathological analysis indicated extensive tumor necrosis in the P407-DOX-SSD-Lps group, demonstrating superior antitumor efficacy over other treatments and highlighting a marked synergistic antitumor effect through the co-delivery of SSD and DOX. Serum biochemical and histopathological analyses confirmed that neither P407-SSD-Lps nor P407-DOX-SSD-Lps induced significant toxicity in major organs of mice. Moreover, they effectively alleviated hepatocellular carcinoma-induced abnormal liver and kidney function. This hepatorenal protective effect may be associated with the anti-inflammatory, antioxidant, and autophagy-regulating activities of SSD (Yu et al., 2021).

Liposomes fabricated exclusively from phospholipids exhibit poor stability, as their phospholipid bilayer organization, particle size, and surface characteristics are susceptible to temporal variations. To address this, researchers typically incorporate lipid components into the phospholipid bilayer to effectively modulate membrane fluidity and enhance physical stability. For instance, the doxorubicin liposomal formulation (Doxil) and amphotericin B (Ambisome) both utilize cholesterol as a stabilizer for treating cancer and other diseases. However, the presence of cholesterol in these liposomes may lead to cardiopulmonary-related adverse effects (Moein Moghimi et al., 2008; Szebeni et al., 2000). In this study, SSD, which shares a analogous steroidal core structure, is utilized to replace cholesterol in liposome preparation, thereby effectively circumventing the potential side effects associated with cholesterol. As a natural anticancer agent, SSD promotes autophagy and mitochondrial function, subsequently upregulating the expression of organic anion transporting polypeptides and reducing tumor extracellular matrix deposition, which collectively enhance drug distribution at tumor sites (Xu et al., 2021). Following tumor accumulation and SSD release, SSD specifically interacts with cholesterol in the tumor cell membrane, perturbing the orderly arrangement of membrane phospholipids, increasing membrane permeability, facilitating intracellular drug transport, and synergistically augmenting the overall anti-hepatocellular carcinoma efficacy (Sudji et al., 2015). In contrast, DOX primarily acts by binding to topoisomerase II in tumor cell DNA to form a stable ternary complex, impeding DNA strand break repair and inducing irreversible double-strand damage. Concurrently, its planar aromatic ring structure intercalates into DNA double helices, altering their spatial conformation and thereby dualistically disrupting DNA replication and transcription processes, ultimately leading to cell cycle arrest (Schurmann et al., 2021). Consequently, the synergistic antitumor effect observed with P407-DOX-SSD-Lps co-delivering SSD and DOX likely arises from their complementary mechanisms of action.

The “drug-excipient integration” strategy in this study not only streamlined the composition of the liposomal formulation but also enhanced therapeutic efficacy by leveraging the inherent antitumor activity of SSD. Moreover, through modification with PA and P407 and the co-delivery of DOX, it achieved multifunctional, multi-pathway synergistic therapy for hepatocellular carcinoma. Compared to conventional chemotherapy, this approach aims to elevate local drug concentrations within tumors while minimizing adverse effects, aligning with contemporary design trends in multifunctional nanomedicine systems (Liang et al., 2024; Peng et al., 2025). Although this delivery system demonstrates significant potential in treating hepatocellular carcinoma, the complexity and heterogeneity of HCC pathogenesis and progression present several challenges that must be addressed for successful clinical translation (Liu et al., 2024). For instance, the manufacturing process and quality control of liposomes still require careful optimization. Particular attention must be paid to the hemolytic toxicity of SSD, and both the actual dosage and potential side effects from prolonged use need thorough evaluation during clinical translation. Therefore, this multi-component delivery system demands rigorous characterization and quality control to meet regulatory standards for nanomedicines, with comprehensive safety evaluation being crucial.

The delivery system developed in this study, while currently applied for co-delivering DOX against orthotopic hepatocellular carcinoma in mouse models, demonstrates potential for constructing broader therapeutic combinations. By leveraging SSD's inherent antitumor activity in conjunction with suitable drugs, it could enhance therapeutic efficacy against diverse tumor types. Subsequent investigations will address existing limitations through systematic refinements. For instance, determining the optimal synergistic ratio between SSD and DOX requires further screening and validation. Additionally, although we evaluated the tumor-targeting capability of P407-SSD-Lps using DiR-loaded formulations in orthotopic liver cancer models, the fluorescence signals from mouse hair and skin tissues during live imaging overlapped with tumor signals, compromising accurate assessment of tumor-specific accumulation. This limitation would be expected to be resolved by employing activatable fluorescent probes that only emit signals upon encountering the specific tumor microenvironment, enabling efficient diagnosis and monitoring of liver cancer (Men et al., 2025). Furthermore, as SSD plays a pivotal role in tumor targeting, multi-omics approaches should be implemented to identify key pathways and molecular targets, thereby elucidating its underlying mechanism of action (Zheng et al., 2024).

## 5 Conclusion

In summary, this study developed a novel multifunctional liposomal delivery system centered on SSD, embodying the “drug-excipient integration” concept. The SSD functioned as a versatile membrane material that formed structurally stable liposomes when combined with SPC and PA. Crucially, it concurrently served as an active pharmaceutical ingredient exerting antitumor effects. Surface modification with P407 not only enhanced biocompatibility but also reinforced structural integrity. The P407-SSD-Lps, prepared using SSD, exhibited good stability, favorable hemocompatibility, superior hepatocellular carcinoma-targeting capability and potent antitumor activity in vitro. Furthermore, in murine orthotopic hepatocellular carcinoma transplantation models, the P407-SSD-Lps demonstrated exceptional tumor-targeting efficacy and significantly suppressed tumor growth. When delivering DOX, P407-DOX-SSD-Lps exerted superior therapeutic effects compared to Free DOX, Free SSD, and P407-SSD-Lps alone, mediated by synergistic interactions between SSD and DOX. Concurrently, both P407-SSD-Lps and P407-DOX-SSD-Lps substantially mitigated cardio-hepato-renal injury in tumor-bearing mice while exhibiting favorable biocompatibility profiles. These results indicated that P407-SSD-Lps represented a promising safe and efficient drug delivery system. By eliminating the cholesterol dependency inherent to conventional liposomes, this work pioneered a “drug-excipient integration” strategy with significant potential for advancing hepatocellular carcinoma therapeutics.

## CRediT authorship contribution statement

**Kun Yu:** Conceptualization, Data curation, Investigation, Writing – original draft, Methodology. **Sicheng Huang:** Investigation, Conceptualization**. Guochun Yang:** Investigation, Writing – original draft. **Junze Tang:** Investigation, Data curation. **Xiaoyu Zhao:** Investigation, Methodology. **Rui Pan:** Investigation. **Hailiang Zhang:** Investigation. **Menghan Xu:** Investigation. **Xiaofei Li:** Resources. **Xin Cheng:** Funding acquisition, Project administration, Supervision, Writing – review & editing. **Anguo Hou:** Funding acquisition, Project administration, Supervision, Writing – review & editing.

## Funding

This work was supported by the 10.13039/501100001809National Natural Science Foundation of China [No. 82560842] and programs of the Yunnan Provincial Department of Education Science Research Fund Project [No. 2024Y345, 2023Y0453]. Yunnan Key Laboratory of Dai and Yi Medicines [No. 2024SS24075]. 12th Five-year Key Construction Discipline of State Administration of Traditional Chinese Medicine “Dai Pharmacy”. Yunnan Provincial Science and Technology Department-Applied Basic Research Joint Special Funds of Chinese Medicine [No. 202001AZ070001-008]. Social development special projects-Key research and development plan of Science and Technology Department of Yunnan Province [No.202303AC100025].

## Declaration of competing interest

The authors declare that they have no known competing financial interests or personal relationships that could have appeared to influence the work reported in this paper.

## Data Availability

Data will be made available on request.

## Appendix A Supplementary data

Supplementary material 1 Image 1

Supplementary material 2 Image 2

Supplementary material 3 Image 3

Supplementary material 4 Image 4
