# Supplementary material for: Multifunctional saikosaponin D-liposomes for hepatocellular carcinoma: Formulation optimization, characterization, and in vitro/in vivo evaluation
Source: Int J Pharm X. 2025 Nov 11;10:100445. doi: 10.1016/j.ijpx.2025.100445 (PMC12664412; doi:10.1016/j.ijpx.2025.100445)
Supplement: Supplementary file 1 — Supplementary material 1 [file mmc1.docx]

**Supplemental Data**

**Multifunctional saikosaponin D-liposomes for hepatocellular carcinoma: Formulation optimization, characterization, and *in vitro/in vivo* evaluation**

Kun Yu ^a,b,c^, Sicheng Huang ^a^, Guochun Yang ^a,b,c^, Junze Tang ^a,c^, Xiaoyu Zhao ^a,c^, Rui Pan ^a,c^, Hailiang Zhang ^a,c^, Menghan Xu ^a^, Xiaofei Li ^a,c^, Xin Cheng ^a,b,c,d,*^, Anguo Hou ^a,b,c,d,*^

^a^ *College of Traditional Chinese Medicine, Yunnan University of Chinese Medicine, Kunming 650500, China*

^b^ *Yunnan Key Laboratory of Dai and Yi Medicines, Kunming 650500, China*

^c^ *Laboratory Animal Center, Yunnan University of Chinese Medicine, Kunming 650500, China.*

^d^ *The Key Laboratory of External Drug Delivery System and Preparation Technology in University of Yunnan Province, Kunming 650500, China*

^*^ Corresponding authors.

Correspondence to: Xin Cheng, College of Traditional Chinese Medicine, Yunnan University of Chinese Medicine, Kunming 650500, China.

*Email addresses*: chengxin920@126.com (Xin Cheng), 1324491101@qq.com (Anguo Hou).

## **Analysis method of SSD**

The concentration of SSD was quantified using high-performance liquid chromatography (HPLC, Agilent Technologies, Inc., USA) at 210 nm. A reversed-phase ZORBAX Eclipse XDB-C18 analytical column (5 µm, 250 × 4.6 mm) was employed with a mobile phase consisting of acetonitrile and water at a ratio of 45:55 (v/v). The flow rate was maintained at 1 mL/min, and the injection volume was 10 µL. The reliability of the HPLC method was validated by assessing specificity, linearity, precision, repeatability, stability, and recovery.

**

**

**Fig. S1.** Method validation. (**a**) Evaluation of specificity. (**A**) P407-SSD-LPs sample solutions, (**B**) SSD standard solutions, (**C**) Excipients solutions, (**D**) Methanol solvent. 1, SSD. (**b**) Standard curve of SSD.

Chromatographic analysis revealed symmetrical SSD peaks with baseline resolution from other components, confirming no interference from liposomal excipients in SSD quantification (Fig. S1a). A calibration curve was established by plotting SSD peak area (*Y*) against concentration (*X*, 5-100 μg/mL) (Fig. S1b). The linear regression equation $\text{Y}\text{ = 3.307}\text{X}\text{–}\text{2.2662}$ (R² = 0.999 4) demonstrated excellent linearity. Precision, repeatability, and stability tests yielded relative standard deviations (RSD) < 2%. Furthermore, SSD recovery rates ranged from 95% to 105% (RSD < 2%), validating the reliability and accuracy of the analytical protocol.

## **Encapsulation efficiency and** **drug loading**

The free SSD was separated from liposomes via ultracentrifugation. The quantities of free and total SSD were quantified using the HPLC analytical method described in the Supplemental Data, from which encapsulation efficiency (*EE*) and drug loading (*DL*) were calculated. Briefly, the liposomal dispersion was transferred into centrifuge tubes and ultracentrifuged at 50,000 rpm for 1 hour at 4°C. The supernatant was precisely aspirated, diluted with methanol, and analyzed to quantify unencapsulated SSD. An equivalent volume of the original liposomal solution was similarly diluted with methanol to determine total SSD content. *EE* and *DL* were calculated using Equations (S1) and (S2), respectively.

$\text{EE}\text{ (\%) = 1}\text{–}\text{ (}\frac{\text{W}\text{1}}{\text{W}\text{2}}\text{) ×100\%}$ (S1)

$\text{DL}\text{ (\%) = }\frac{\text{(}\text{W}\text{2 }\text{–}\text{ }\text{W}\text{1}\text{)}}{\text{W}\text{3}}\text{×100\%}$ (S2)

where *W*_1_ denotes the mass of free SSD in the liposomal solution, *W*_2_ represents the total mass of SSD, and *W*_3_ corresponds to the total mass of liposomal materials.

## **Single-factor experiment**

In the single-factor experiments, six factors were evaluated: the mass of SPC, water bath temperature, hydration time, and the mass ratios of SPC to SSD (w/w), PA (w/w), and P407 (w/w). These factors were examined across four levels. The single-factor experimental design is shown in Table S1.

**Table S1** Levels of experimental factors.

| The mass of SPC (mg) | SPC:SSD | SPC:PA | SPC:P407 | Water bath temperature (℃) | Hydration time (min) |
| --- | --- | --- | --- | --- | --- |
| 10 | 10:1 | 6:1 | 2:1 | 40 | 15 |
| 20 | 12:1 | 8:1 | 4:1 | 45 | 20 |
| 30 | 15:1 | 10:1 | 6:1 | 50 | 25 |
| 40 | 20:1 | 12:1 | 8:1 | 55 | 30 |





**Fig. S2.** Effects of six factors on liposome characteristics. (**a**) Effects of different SPC masses on liposome characteristics. (**b**) Effects of SPC to SSD ratios on liposomal properties. (**c**) Effects of SPC to PA ratios on liposomal properties. (**d**) Effects of SPC to P407 ratios on liposomal properties. (**e**) Effects of hydration temperature on liposomal properties. (**f**) Effects of hydration time on liposomal properties.

Fig. S2 illustrates the effects of six critical factors on liposomal characteristics. Under all tested conditions, the liposomes maintained particle sizes below 150 nm with PDI values under 0.3, demonstrating consistently favorable monodisperse size distribution and homogeneous dispersion across the experimental ranges. Notably, while these fundamental physical properties remained stable, three key parameters including the mass of SPC, SPC/SSD ratio, and SPC/P407 ratio exerted pronounced effects on both encapsulation efficiency and zeta potential. These factors were therefore selected for subsequent orthogonal optimization experiments.

**Table S2** Influential factors and levels of L_9_ (3^4^) orthogonal experiment.

| Level | The mass of SPC (A) | SPC:SSD (B) | SPC:P407 (C) |
| --- | --- | --- | --- |
| 1 | 25 | 11:1 | 2:1 |
| 2 | 30 | 12:1 | 4:1 |
| 3 | 35 | 14:1 | 6:1 |

## **In vitro release testing**

The *in vitro* release profiles of free SSD and P407-SSD-Lps were investigated using a dialysis method. Phosphate-buffered saline (PBS) containing 0.5% polysorbate 80 served as the release medium. Aliquots of free SSD solution and P407-SSD-Lps were placed into dialysis bags (molecular weight cutoff: 8000 Da), sealed, and immersed in the release medium under constant agitation (100 rpm) at 37°C. Samples were collected at 1, 2, 4, 8, 12, 24, 48, and 72 hours, with equal volumes of pre-warmed fresh medium replenished after each sampling. The SSD content was quantified according to the “Supplementary Section 1” Cumulative release percentage was calculated using Equation. (S3), and release curves were plotted.

$\text{Cumulative released percentage}\text{ (\%)}\text{ }\text{=}\frac{\text{C}_{\text{m}}\text{V+}\sum_{\text{n=1}}^{\text{m-1}} \text{(}\text{C}_{\text{n}}\text{V}_{\text{n}}\text{)}}{\text{W}}\text{×100\%}$ (S3)

where *C*_m_ and *C*_n_ denote the concentrations of SSD in the release medium at the m-th and n-th sampling time points, respectively; *V* represents the total volume of the release medium, and *V*_n_ corresponds to the volume withdrawn at the n-th sampling. *W* indicates the mass of SSD initially loaded.

**Table S3** Model fitting results of P407-SSD-LPs drug release.

| Fitting models | Fitting equation | R^2^ |
| --- | --- | --- |
| Zero-Order | $\text{Q}_{\text{t}}\text{=0.6581 }\text{t}\text{+22.2933}$ | 0.7246 |
| First-Order | $\text{Q}_{\text{t}}\text{=57.6196 (1}\text{–}\text{e}^{-\text{0.1133}\text{t}}\text{)}$ | 0.9775 |
| Higuchi | $\text{Q}_{\text{t}}\text{=7.4266}{\text{ }\text{t}}^{\text{1/2}}\text{+6.4766}$ | 0.8967 |
| Ritger-Peppas | $\text{Q}_{\text{t}}\text{=0.9466}{\text{ }\text{t}}^{\text{0.}\text{4174}}$ | 0.9408 |
| Weibull | $\text{Q}_{\text{t}}\text{=}\text{100 (1–}\text{e}^{{-\text{(0.0151(}\text{t}\text{–}\text{0.4851))}}^{\text{0.4318}}}\text{)}$ | 0.9848 |

## **Stability**

P407-SSD-Lps were prepared according to the optimized formulation and preparation process, then stored at 4°C. *EE*, particle size, PDI, and zeta potential were measured at 1, 7, 15, and 30 days to evaluate their stability.

**Table S4** Results of Stability Experiments (mean ± SD, n=3).

| Time (d) | EE (%) | Particle size (nm) | PDI | Zeta potential (mV) |
| --- | --- | --- | --- | --- |
| 1 | 80.45±1.29 | 127.68±2.35 | 0.205±0.011 | –50.06±1.14 |
| 7 | 80.14±1.39 | 126.37±2.07 | 0.212±0.012 | –48.24±1.52 |
| 15 | 79.90±1.03 | 133.19±1.21 | 0.219±0.008 | –46.25±1.98 |
| 30 | 78.80±1.12 | 139.59±3.12 | 0.241±0.012 | –44.65±1.27 |

## **Hemolysis evaluation**

The fresh mouse blood was collected into heparin-coated centrifuge tubes and centrifuged at 2500 rpm for 5 minutes at 4°C to remove the plasma. The erythrocytes were gently resuspended in normal saline, centrifuged at 2000 rpm for 7 minutes, and the supernatant discarded. This washing procedure was repeated three times. The erythrocytes were diluted with normal saline to prepare a 2% erythrocyte suspension (v/v). Free SSD and P407-SSD-Lps were diluted to SSD concentrations of 1.0, 2.0, 3.0, 5.0, and 8.0 μg/mL using normal saline. Aliquots of the sample solutions were mixed with the 2% erythrocyte suspension in centrifuge tubes and incubated at 37°C for 3 hours. After incubation, the mixtures were centrifuged at 2000 rpm for 15 minutes, and the absorbance (*A*) of the supernatant was measured at 540 nm using a UV-Vis spectrophotometer (Agilent Technologies, Inc., USA) (Demirturk et al., 2024)^.^ The hemolytic index was calculated using Equation (S4).

$\text{Hemolytic index}\text{ (\%) = }\frac{\text{(}\text{A}\text{test }\text{–}\text{ }\text{A}\text{negative control}\text{)}}{\text{(}\text{A}\text{positive control }\text{–}\text{ }\text{A}\text{negative control}\text{)}}\text{×100\%}$ (S4)


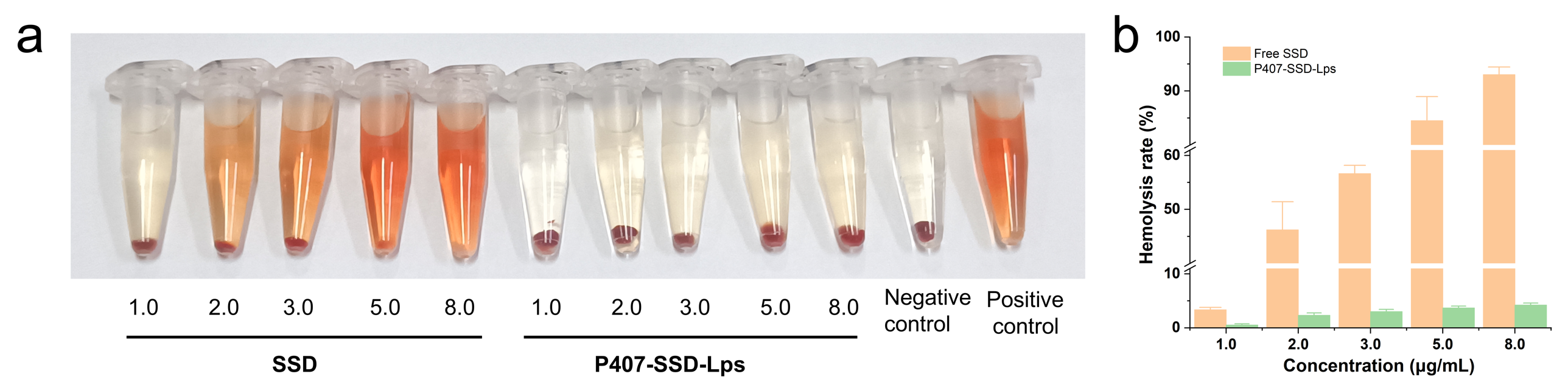


**Fig. S3.** (**a**) Incubation outcomes of 2% erythrocyte suspension with varying concentrations of Free SSD and P407-SSD-Lps. (**b**) Hemolysis rates post 2 hours incubation (mean ± SD, n=3).

As shown in Fig. S3, free SSD at 2 μg/mL induced a hemolysis rate of (46.20 ± 5.23)% (hemolysis >5% indicates significant erythrocyte damage). In contrast, P407-SSD-Lps exhibited hemolysis rates < 5% across the tested concentration range (1-8 μg/mL), with mean values of (0.54 ± 0.21)%, (2.31 ± 0.47)%, (3.10 ± 0.40)%, (3.68 ± 0.34)%, and (4.22 ± 0.37)%, respectively. These results confirmed the excellent hemocompatibility of the P407-SSD-Lps system.

## ***In vitro* targeting**

The P407-DiR-Chol-Lps and P407-DiR-SSD-Lps were prepared by encapsulating the near-infrared fluorescent probe DiR into liposomes using the optimized formulation and process. The *in vitro* targeting capability of these liposomal systems was subsequently evaluated based on their cellular uptake by HepG2 cells. For this purpose, HepG2 cells were seeded in 12-well plates at 3×10^5^ cells/well and cultured for 24 hours. The cells were then treated with either P407-DiR-Chol-Lps or P407-DiR-SSD-Lps. After 6 hours and 12 hours of incubation, residual fluorescent dye was removed by washing with PBS. Cellular uptake was visualized under an inverted fluorescence microscope, and semi-quantitative analysis was performed using Image J software to quantify intracellular DiR fluorescence intensity across experimental groups.


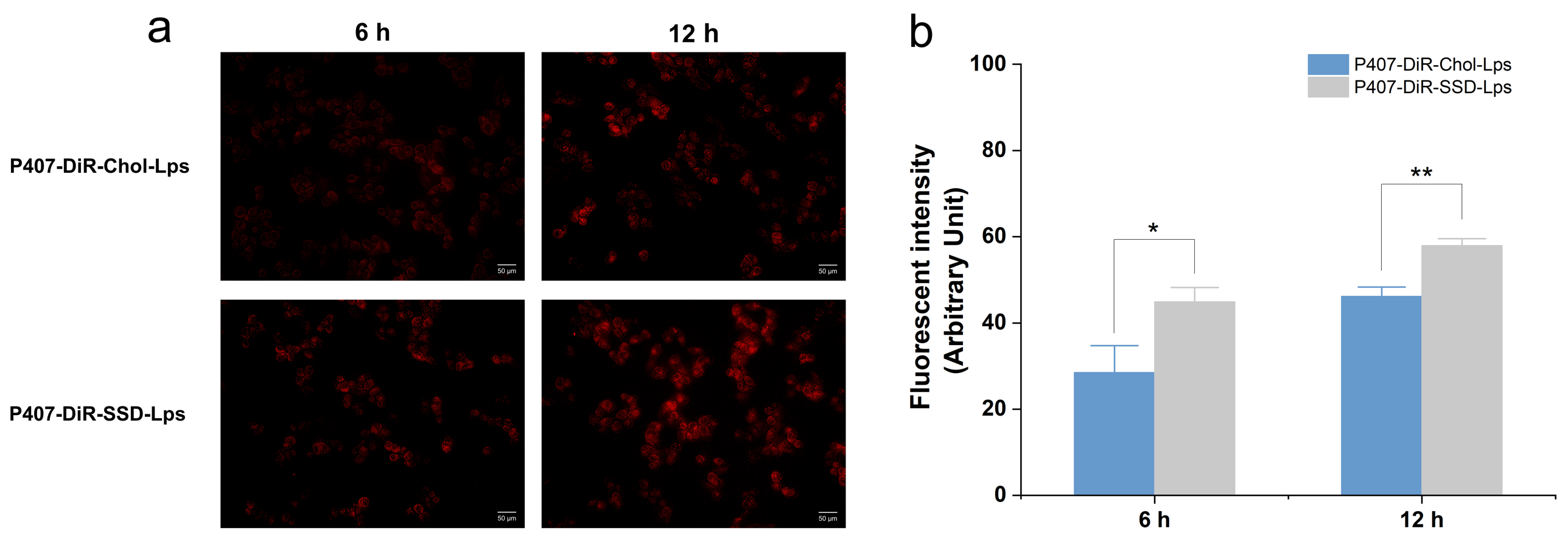


**Fig. S4.** (**a**) Cellular uptake of P407-DiR-Chol-Lps and P407-DiR-SSD-Lps by HepG2 cells. The images were obtained at 200× magnification (scale bar=50 µm). Red fluorescence indicates internalized DiR within cells. (**b**) The fluorescence intensity of P407-DiR-Chol-Lps group and P407-DiR-SSD-Lps group was compared (mean ± SD, n=3).

**Note:** In figure (**b**), ^*^ p < 0.05, ^***^ p < 0.001.

Fig. S4a demonstrates that the P407-DiR-SSD-Lps group exhibited stronger fluorescence intensity than the P407-DiR-Chol-Lps group at both 6 hours and 12 hours post-incubation. Semi-quantitative analysis using ImageJ software showed fluorescence intensities of (44.92 ± 3.34) AU at 6 hours and (57.91±1.64) AU at 12 hours for the P407-DiR-SSD-Lps group, which were significantly higher than those of the P407-DiR-Chol-Lps group (28.49 ± 6.24) AU and (46.16 ± 2.18) AU (Fig. S5b). This finding demonstrated that the P407-DiR-SSD-Lps enhanced the uptake efficiency of HepG2 cells, potentially attributable to the interaction between SSD with Chol in cellular lipid membranes. This interaction altered phospholipid arrangement, enhanced membrane permeability, and thereby promoted DiR delivery (Sudji et al., 2015).

## **Inhibition of hemorrhagic ascites and abdominal wall tumors**

Post-mortem examination revealed severe abdominal adhesions in 85.7% of the model group mice, whereas all treatment groups exhibited significantly reduced adhesion severity. Notably, the P407-DOX-SSD-Lps group showed optimal therapeutic outcomes, with 57.1% of mice were adhesion-free, with no severe adhesions observed in the remaining subjects (Table S5). The model group developed substantial hemorrhagic ascites, the volume was measured at (9.014 ± 1.120) mL, indicating extensive tumor infiltration. In contrast, the free SSD and P407-SSD-Lps groups demonstrated significantly lower ascites incidence and reduced volumes, it was recorded as (5.380 ± 1.438) mL and (2.467 ± 1.079) mL, respectively. Neither the free DOX nor P407-DOX-SSD-Lps groups showed ascites formation. Compared to the model group, all treatment groups exhibited significantly reduced incidence and weight of abdominal wall tumors. The model group showed abdominal wall tumor weights of (1.427 ± 0.205) g. In the free DOX group, the weight of abdominal wall tumor was (0.382 ± 0.063) g. The free SSD group had an abdominal wall tumor weight of (0.820 ± 0.087) g, and the P407-SSD-Lps group had an abdominal wall tumor weight of (0.419 ± 0.037) g. Remarkably, the P407-DOX-SSD-Lps group demonstrated superior efficacy and the abdominal wall tumor incidence was only 42.9%, with abdominal wall tumor weight significantly reduced to (0.166 ± 0.022) g (p < 0.05, Table S6).

**Table S5** Comparison of abdominal adhesion grade among the five groups (mean ± SD, n=7).

| Adhesion Grade | Model | Free DOX | Free SSD | P407-SSD-Lps | P407-DOX-SSD-Lps |
| --- | --- | --- | --- | --- | --- |
| Lv. 0 | 0% | 42.9% | 0% | 28.6% | 57.1% |
| Lv. 1 | 0% | 42.9% | 28.6% | 42.9% | 28.6% |
| Lv. 2 | 14.3% | 14.3% | 57.1% | 28.6% | 14.3% |
| Lv. 3 | 42.9% | 0% | 14.3% | 0 % | 0% |
| Lv. 4 | 42.9% | 0% | 0% | 0% | 0% |

**Table S6** Comparison of tumor status among the five groups (mean ± SD, n=7).

| Group | Ascites Occurrence  Rate (%) | Ascites  Volume (mL) | AW Tumor  Occurrence Rate (%) | AW Tumor  Weight (g) |
| --- | --- | --- | --- | --- |
| Model | 100 | 9.014±1.120 | 100 | 1.427±0.205 |
| Free DOX | 0 | 0 ^***##&&&^ | 57.1 | 0.382±0.063 ^***###^ |
| Free SSD | 71.4 | 5.380±1.438 ^*^ | 71.4 | 0.820±0.087 ^**^ |
| P407-SSD-Lps | 42.9 | 2.467±1.079 ^*#^ | 57.1 | 0.419±0.037 ^***##^ |
| P407-DOX-SSD-Lps | 0 | 0 ^***##&&&^ | 42.9 | 0.166±0.022 ^***###&&†^ |

**Note:** vs Model group, ^*^ p < 0.05, ^**^ p < 0.01, ^***^ p < 0.001. vs Free SSD group, ^##^ p < 0.01, ^###^ p < 0.001. vs P407-SSD-Lps group, ^&&^ p < 0.01, ^&&&^ p < 0.001. vs Free DOX group, ^†^p < 0.05.

# **References**

Demirturk, N., Varan, G., Kaga, S., Malanga, M., Bilensoy, E., 2024, Optimization and characterization of Rituximab targeted multidrug loaded cyclodextrin nanoparticles against Non-Hodgkin Lymphoma. INT J PHARMACEUT 662, 124488.

Sudji, I., Subburaj, Y., Frenkel, N., García-Sáez, A., Wink, M., 2015, Membrane Disintegration Caused by the Steroid Saponin Digitonin Is Related to the Presence of Cholesterol. MOLECULES 20, 20146-20160.
