# Supplementary material for: Multifunctional saikosaponin D-liposomes for hepatocellular carcinoma: Formulation optimization, characterization, and in vitro/in vivo evaluation
Source: Int J Pharm X. 2025 Nov 11;10:100445. doi: 10.1016/j.ijpx.2025.100445 (PMC12664412; doi:10.1016/j.ijpx.2025.100445)
Supplement: Supplementary file 2 — Supplementary material 2 [file mmc2.pdf]

# 云南中医药大学动物实验福利与伦理审查表（供项目实施）

**Yunnan University of Chinese Medicine Application Format for Welfare and Ethical Approval for Research Involving Animals**

申请日期：2025 年 06 月 18 日　批准文号：YNUTCM-XMSS-G-20250051
Appl. Date　Y　M　D　IACUC Issue No.

申请的相关信息 Related Information Filled by Applicant

| | | | | |
|---|---|---|---|---|
| 课题名称：基于柴胡皂苷 D 构建的多功能脂质体设计、表征及体内外抗肝癌疗效评价<br>Program | | | | |
| 课题来源：云南省教育厅科学研究基金<br>Sponsor | | 项目代码：11370104828<br>Funding code | | |
| 课题级别：Grade | □国家级 National level | ☑ 省级 Provincial level | □厅级 Department level | □其他：________ Other |
| 课题负责人：喻锟<br>Name of PI | | 部门及电话：中药学院/18314213078<br>Department & Tel. | | 邮箱：2874553723@qq.com<br>E-mail |
| 动物实验负责人（联系人）：喻锟<br>Director of animal experiment (Contact person) | | 部门及电话：中药学院/18314213078<br>Department & Tel. | | 邮箱：2874553723@qq.com<br>E-mail |
| 实验动物从业资格证<br>Qualification certificate for laboratory animal research | 姓名<br>Name | 证书编号<br>Certificate No. | 截止日期<br>Expire date | 发证机关<br>Issuing unit |
| | 喻锟 | ZRXK20240255 | 2026-11-18 | 云南中医药大学动物实验中心 |
| | | | | |
| | | | | |
| | | | | |
| | | | | |
| 实验动物来源（采购单位名称）：云南致力科技有限公司<br>Source of laboratory animal (Procurement) | | 采购地生产许可证编号：SCXK（京）2024-0001<br>Certification number of production | | |
| 采购地质量合格证：Certification of fitness　☑ 有 Yes　□ 无 No | | 本单位动物实验设施许可证编号：SYXK（滇）K2022-0004<br>Certification number of the Facilities | | |
| 品种/品系：Breed/Strain<br>□ 大鼠(Rat)：________<br>☑ 小鼠(Mouse)：KM<br>□ 豚鼠(Guinea pig)：________<br>□ 兔(Rabbit)：________<br>□ 实验鱼(Fish)：________<br>□ 其他(Other)：______ | | 等级：Grade<br>□ 普通(CV)<br>□ 清洁(CL)<br>☑ SPF<br>□ 无菌级(GF) | | |
| 数量：Number　♂ 60 只、♀ 0 只；总共(Total) 60 只 | | 周/月龄：4 周龄<br>W/M Age | 体重(g)：18-22<br>Weight (g) | |

拟开展实验时间： 2024 年 12 月 15 日 至 2026 年 07 月 01 日
Experimental period Y M D to Y M D

动物实验项目的目的、必要性、意义、方法步骤、主要观察指标
Experimental objective, necessity, significance, method and how the program has been designed to achieve the objectives of the research，main observation target

1. 实验目的：研究基于柴胡皂苷 D 构建的多功能脂质体体内外抗肿瘤作用等。
2. 必要性：将经典的中医药柴活性成分与新型给药系统相结合构建新型多功能化载体，该载体可降低药物毒性，提高生物利用度和实现对病灶部位的精准靶向。(1)不能被非活体实验替代原因:研究过程中需昆明种小鼠造模研究柴胡皂苷 D 脂质体对原发性肝癌的治疗作用和靶向性。因此，不可被非活体实验替代。(2)不可被无伤害人体实验替代原因:本实验造模需要将鼠肝癌细胞打入鼠肝脏造成肝癌模型,造成较严重疾病才可以进行实验研究，因此，不可被无伤害人体实验替代。本实验可为原发性肝癌治疗提供新的思路，为后续实验研究奠定坚实基础。
3. 意义：植物药是人类与疾病数千年斗争的支柱，在临床治疗中占重要地位。其作为原发性肝癌的一种治疗方式，具有低毒高效、靶点多样等优势。将其与新型给药系统相结合研究，探索如何实现对疾病的精准治疗有着重要的意义。
4. 方法步骤：柴胡皂苷构建的脂质体对小鼠原发性肝癌的体内药效及靶向性研究
   1）昆明种小鼠体内药效研究。分为 6 个组：空白组、模型组、游离柴胡皂苷治疗组、阿霉素阳性药组、柴胡皂苷脂质体治疗组、阿霉素-柴胡皂苷脂质体治疗组。每组需要 8 只。
   步骤：采用 H22 细胞注射至小鼠肝脏部位造模形成原位肝癌模型，以腹腔注射方式隔天给药，在第 5、11、16 天时，采用 b 超观察肿瘤生长情况，第 17d 天时采用浓度为 2%的异氟烷麻醉腹主动脉取血，安乐死后，取脏器和肿瘤等。
   2）柴胡皂苷脂质体肝脏靶向性研究。分为 2 个组：DIR-柴胡皂苷脂质体组、DIR-胆固醇脂质体组。每组需昆明种小鼠 8 只。
   步骤：采用 H22 细胞注射至小鼠肝脏部位造模形成原位肝癌模型，以尾静脉注射荧光探针，使用小动物活体成像仪器观察不同时间点活体动物荧光分布情况，采用浓度为 2%的异氟烷麻醉安乐死后取肿瘤、脏器进行荧光成像观察荧光分布及强度。

5. 主要观察指标：肿瘤成瘤情况，小鼠和大鼠进食、体重变化，生存期考察，肿瘤和脏器指数，进行磷脂酰肌醇蛋白聚糖 3（GPC-3）、甲胎蛋白（AFP）、癌胚抗原（CEA）、丙氨酸氨基转移酶（ALT），天门冬氨酸氨基转移酶（AST），碱性磷酸酶（AIP/AKP）和γ-谷氨酰氨基转移酶（γ-CT）活性等血清生化检测，心、肝、脾、肺、肾、脑、肿瘤等组织病理检测，ELISA 法检测血清肿瘤坏死因子-α（TNF-α）和白细胞介素-6（IL-6）、白细胞介素-1β（IL-1β），全血细胞计数，等，进行离体和活体荧光成像，超声影像检测等诊断。

仁慈终点或实验终结的指标
Humane endpoint or experimental terminative indicator
严格遵循“替代、减少、优化”的 3R 准则开展动物实验。进行体内药效实验、生存期考察和靶向性等实验。(1)若实验过程中动物异常需要提前终止动物实验情况:在实验临近实验终点时，若动物即将遭受肝癌或关节炎带来的无法减轻的剧痛和不适，采用仁慈终点替代实验终点。(2)若无异常情况，正常实验终结指标:动物无异常，实验取得希望成果，需要实验终结时采用浓度为 2%

<table>
<tr><td></td><td>的异氟烷麻醉动物后颈椎脱臼致死，取脏器、肿瘤等组织。</td></tr>
<tr><td></td><td>动物死亡处理<br>Death conduct<br><br>□ $CO_2$ 窒息<br>$CO_2$ Suffocated<br><br>□ 麻醉后放血致死<br>Exsanguinations with anesthesia<br><br>□ 麻醉过量致死<br>Anesthesia overdose<br><br>☑ 麻醉后颈椎脱臼致死<br>Cervical dislocation with anesthesia<br><br>□ 其他 请详细说明：<br>Other, detailed description</td></tr>
<tr><td>申请者声<br>Declaration of Applican</td><td>声明：<br>1. 我将自觉遵守实验动物福利伦理相关法规和各项规定，同意接受伦理委员会和实验动物管理者的监督与检查；<br>2. 本人保证申请表中所填内容真实、详尽和易懂。<br><br>Declaration:<br>1. I will abide by the law and regulation stipulation, and accept the supervision and inspection by the committee and laboratory animal department.<br>2. The information I have given is accurate, detailed and comprehensive.<br><br>声明人：课题负责人签（章）： 喻锟<br>Declarant: Signature (stamp) of PI：<br><br>动物实验负责人签（章）： 喻锟<br>Signature (stamp) of Director of Animal Experiment<br><br>2025 年 06 月 18 日<br>Y M D</td></tr>
</table>

| | |
|---|---|
| 审查结果 **Results of Inspection** | 动物实验伦理委员会意见<br>Approval Opinion of the Animal Experimental Ethics Committee<br><br>☑ 批准 Approval　　□ 不批准 Not approve<br><br>云南中医药大学 动物实验伦理审查委员会<br>盖章<br>Stamp<br>2025 年 06 月 19 日<br>Y M D |

填表说明：1．编号由受理部门填写；2.在符合条件的选项前方框中打“√”；2．签名处必须手签、签章或电子签名；3.请准确完整填表，不得涂改，涂改即失效；4.　请双面打印。

# 实验动物使用许可证

（副　本）

许可证号：SYXK（滇）K2022-0004

单位名称：云南中医药大学

法定代表人：丁中涛

此件仅供程欣课题负责人其他使用
指定公司:---
品种:小鼠
有效期2025年07月01日至2025年07月07日
此件仅供在此设施内使用

设施地址：云南中医药大学医鉴甲楼一楼（观察室一至四）、二楼、三楼

适用范围：屏障环境：SPF级大鼠、小鼠

普通环境：普通级兔、豚鼠、实验鱼

有　效　期：2022年7月22日至2027年7月21日

发证单位：昆明市科学技术局

发证日期：2022年11月25日

# 实验动物生产许可证

许可证号：SCXK（京）2024-0001

单位名称：斯贝福（北京）生物技术有限公司

法定代表人：战大伟

设施地址：北京市延庆区八达岭经济技术开发区西康路23号

适用范围：屏障环境：小鼠、大鼠、长爪沙鼠、地鼠、豚鼠；隔离环境：小鼠、大鼠、长爪沙鼠、地鼠、豚鼠；普通环境：饲料、垫料

有效期限：2024年03月27日 至 2029年03月27日

发证机关：北京市科学技术委员会
中关村科技园区管理委员会
（北京市科学技术委员会代章）

发证日期：2024年03月27日

注：此件由北京市科学技术委员会提供，仅供办理政务服务事项时使用，有效期至2029-03-27
