# Supplementary material for: Multifunctional saikosaponin D-liposomes for hepatocellular carcinoma: Formulation optimization, characterization, and in vitro/in vivo evaluation
Source: Int J Pharm X. 2025 Nov 11;10:100445. doi: 10.1016/j.ijpx.2025.100445 (PMC12664412; doi:10.1016/j.ijpx.2025.100445)
Supplement: Supplementary file 3 — Supplementary material 3 [file mmc3.pdf]

细胞遗传质量鉴定检测

Cell Line Authentication Service

STR 基因型检测报告

送检单位：镜像绮点

检品名称：细胞系

检测单位：上海翼和应用生物技术有限公司

报告日期：2024-05-29

Biowing Biotechnology, Shanghai, China 邮箱：zhusn@biowing.com.cn 技术服务咨询：86+18521538068
联系地址：上海市松江区广富林街龙腾路 1015 弄 2 号楼 518 室

报 告 说 明

1. 本报告只对送检的来样负责。
2. 检验报告上的检验结果和检验单位名称，未经同意不得用于广告、评优及商业宣传。
3. 对本报告有异议，请于收到报告之日起十五日内以书面方式提出，逾期不予受理。
4. 对纸质检验报告涂改、增删，或未加盖检验单位印章的复印件均无效。

Biowing Biotechnology, Shanghai, China 邮箱：zhusn@biowing.com.cn 技术服务咨询：86+18521538068
联系地址：上海市松江区广富林街龙腾路 1015 弄 2 号楼 518 室

样 品 信 息

样品编号：

客户样本编号	公司编号
hepG2	20240527-01

样品数量： 1

样品性状： 细胞系

检测项目： STR

送检单位： **镜像绮点**

检测方法： 用 Axygen 的基因组抽提试剂盒提取 DNA，采用 21- STR 扩增方案扩增，在 ABI 3730XL 型遗传分析仪上对 STR 位点和性别基因 Amelogenin 进行检测。

Biowing Biotechnology, Shanghai, China 邮箱：zhusn@biowing.com.cn 技术服务咨询：86+18521538068
联系地址：上海市松江区广富林街龙腾路 1015 弄 2 号楼 518 室

检 测 结 果

(一) 检验基本情况

公司编号	多等位基因	匹配细胞系	细胞库	EV 值	匹配说明
20240527-01	无	GS-HepG2	DSMZ	1.0	完全匹配

样本基因型检验结果

- 多等位基因指三等位及以上基因现象。
- 本次检测各细胞分型结果良好。

(二) 各样本描述

- 20240527-01：该株细胞 DNA 分型在细胞系检索中找到**完全匹配**的细胞系，DSMZ 数据库显示细胞名为 **GS-HepG2**，细胞号对应 **RCB1681**。本次检测在该细胞系中**没有发现多等位基因**。

EV	Cell No.	Cell name	Locus names								
			D5S818	D13S317	D7S820	D16S539	VWA	TH01	AM	TPOX	CSF1PO
	Query (Your Cell)		11,12	9,13	10,10	12,12	17,17	9,9	X,Y	8,9	10,11
1.0(36/36)	RCB1681	GS-HepG2	['11', '12']	['9', '13']	['10', '10']	['12', '12']	['17', '17']	['9', '9']	['X', 'Y']	['8', '9']	['10', '11']
0.94(34/36)	ACC-180	HEP-G2	['11', '12']	['9', '13']	['10', '10']	['12', '13']	['17', '17']	['9', '9']	['X', 'Y']	['8', '9']	['10', '11']

备注：待测细胞系与收录于 ATCC, DSMZ（DSMZ 收录了来自 ATCC、 DSMZ、JCRB 和 RIKEN 等 2490 株细胞的 STR 数据）, ExPASy 细胞库（ExPASy 收录了来自于 ATCC、DSMZ、JCRB、ECACC 和 Riken 等数据库约 8,000 株人源细胞 STR 数据）中的 STR 数据匹配，未收录于上述细胞库的细胞将无法匹配。根据 ATCC 标准委员会鉴定标准（ANSI/ATCC ASN-0002-2022）,匹配度 EV≥80%认为它们具有相关性，可能衍生于共同的祖先细胞；匹配度 55%-80%之间，需要结合其它方法进一步的鉴定认证其相关性。

Biowing Biotechnology, Shanghai, China 邮箱：zhusn@biowing.com.cn 技术服务咨询：86+18521538068
联系地址：上海市松江区广富林街龙腾路 1015 弄 2 号楼 518 室

（三）样本分型结果

细胞的 STR 位点和 Amelogenin 位点的基因分型结果						
Loci	送检细胞 STR 信息			细胞库细胞 STR 信息		
	送检细胞名：hepG2			细胞库细胞名：GS-HepG2		
	Allele1	Allele2	Allele3	Allele1	Allele2	Allele3
D5S818	11	12		11	12	
D13S317	9	13		9	13	
D7S820	10	10		10	10	
D16S539	12	12		12	12	
VWA	17	17		17	17	
TH01	9	9		9	9	
AMEL	X	Y		X	Y	
TPOX	8	9		8	9	
CSF1PO	10	11		10	11	
D12S391	21	25				
FGA	22	25				
D2S1338	19	20				
D21S11	29	31				
D18S51	13	14				
D8S1179	15	16				
D3S1358	15	16				
D6S1043	13	13				
PENTAE	15	20				
D19S433	15.2	15.2				
PENTAD	9	13				
D1S1656	11	12				

Biowing Biotechnology, Shanghai, China 邮箱：zhusn@biowing.com.cn 技术服务咨询：86+18521538068
联系地址：上海市松江区广富林街龙腾路 1015 弄 2 号楼 518 室

其他说明

(一) 分型方案及位点分布

	方案 1	方案 2	方案 3	方案 4
1	D3S1358	D8S1179	D19S433	AMEL
2	VWA	D21S11	TH01	D1S1656
3	D7S820	D16S539	D13S317	D5S818
4	CSF1PO	D2S1338	TPOX	D12S391
5	PENTAE	PENTAD	D18S51	FGA
6			D6S1043	

实验方案及位点

(二) STR 数据库比对

本公司采用 DSMZ tools 进行细胞系比对，其中包含来自于 ATCC, DSMZ, JCRB 和 RIKEN 数据库的 2455 个细胞系 STR 数据。如果待检测细胞未收录于以上细胞库或这是自行建立的新细胞系将无法进行比对，用户需根据细胞分型结果自行与其他数据库进行比对。

(三) 文献引用参考

ATCC SDO. 2011. ASN-0002. Authentication of Human Cell Lines: Standardization of STR Profiling. ANSI eStandards Store, by ATCC-Standards Development Organization (SDO). https://webstore.ansi.org/standards/atcc/ansiatccasn00022011. Accessed: September, 2021.

主要实验人员： 何秀川

复核人: 张晨茜

负责人： 王敏

签发日期：2024-05-29

Biowing Biotechnology, Shanghai, China 邮箱：zhusn@biowing.com.cn 技术服务咨询：86+18521538068
联系地址：上海市松江区广富林街龙腾路 1015 弄 2 号楼 518 室

Applied Biosystems

GeneMapper ID v3.2

20240527

Sample File	Sample Name	Panel	SQO	OS	SQ
32_H04_Cellidentification-1--0528.fsa	hepG2	21Plex_STR_Panel_v1.2			

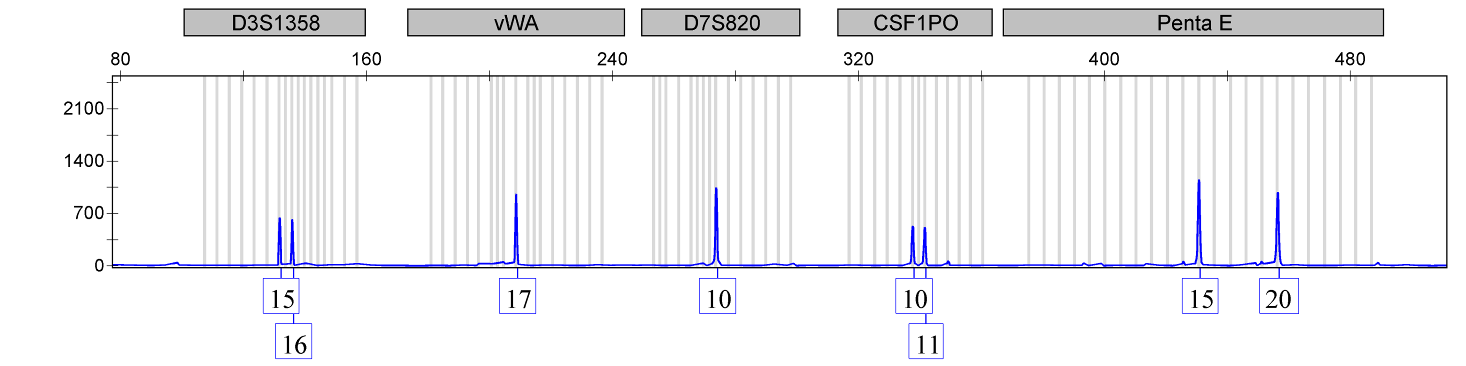

32_H04_Cellidentification-1--0528.fsa hepG2 21Plex_STR_Panel_v1.2

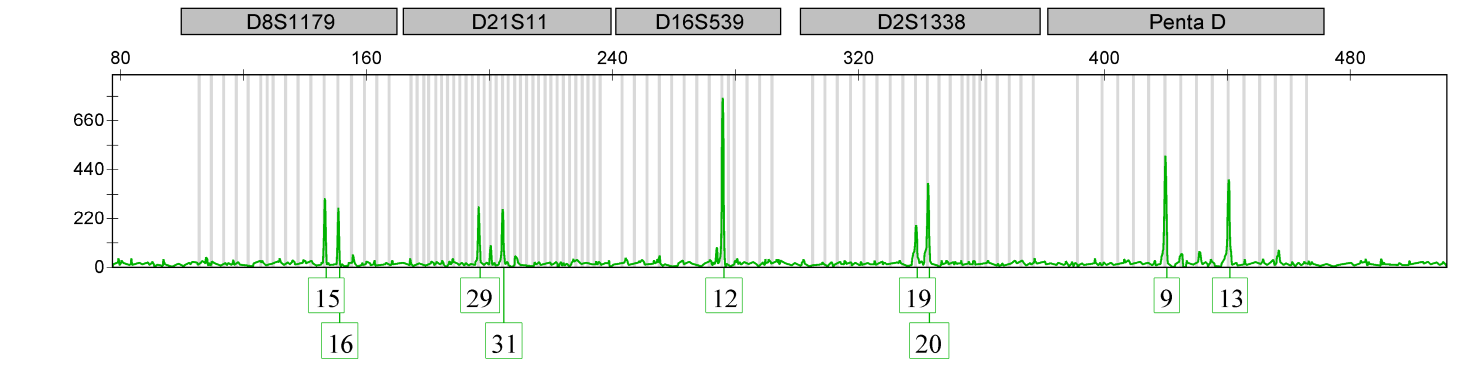

32_H04_Cellidentification-1--0528.fsa hepG2 21Plex_STR_Panel_v1.2

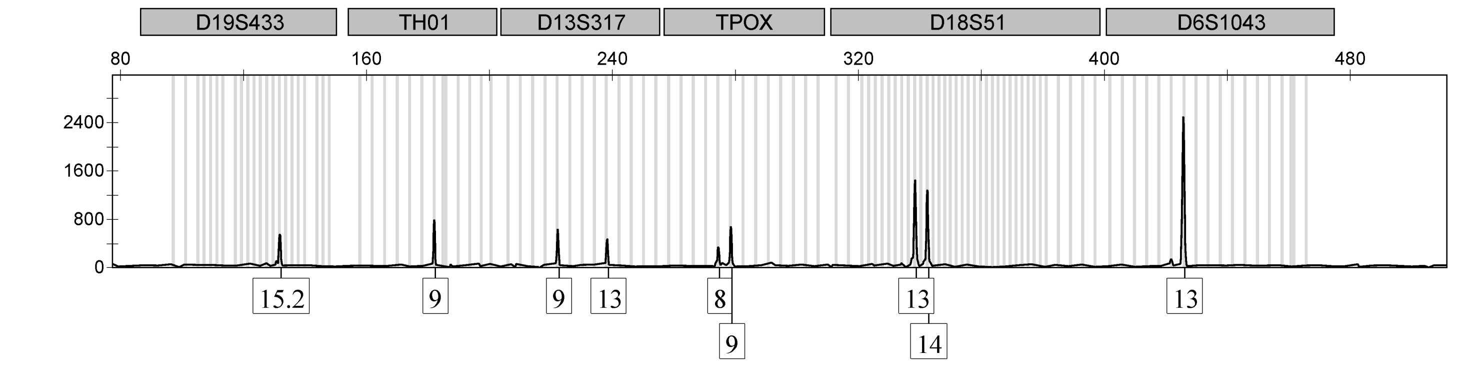

32_H04_Cellidentification-1--0528.fsa hepG2 21Plex_STR_Panel_v1.2

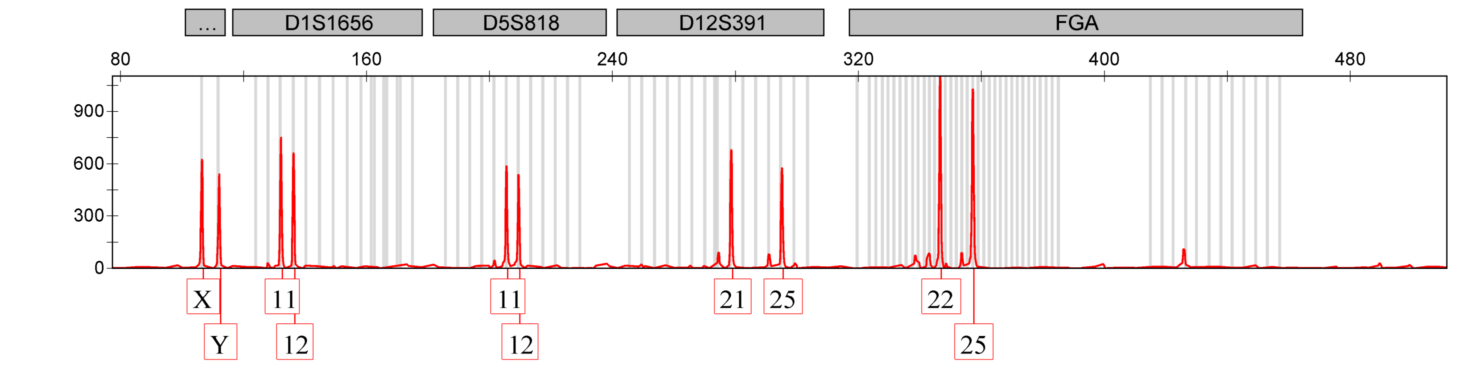

EV	Cell No.	Cell name	Locus names								
			D5S818	D13S317	D7S820	D16S539	VWA	TH01	AM	TPOX	CSF1PO
	Query (Your Cell)		11,12	9,13	10,10	12,12	17,17	9,9	X,Y	8,9	10,11
1.0(36/36)	RCB1681	GS-HepG2	['11', '12']	['9', '13']	['10', '10']	['12', '12']	['17', '17']	['9', '9']	['X', 'Y']	['8', '9']	['10', '11']
0.94(34/36)	ACC-180	HEP-G2	['11', '12']	['9', '13']	['10', '10']	['12', '13']	['17', '17']	['9', '9']	['X', 'Y']	['8', '9']	['10', '11']
1.0(36/36)	CVCL_H214	HepG2-GS	['11', '12']	['9', '13']	['10', '10']	['12', '12']	['17', '17']	['9', '9']	['X', 'Y']	['8', '9']	['10', '11']
1.0(36/36)	CVCL_8560	HepG2-GS-CYP3A4	['11', '12']	['9', '13']	['10', '10']	['12', '12']	['17', '17']	['9', '9']	['X', 'Y']	['8', '9']	['10', '11']
0.94(34/36)	ACC-180	HEP-G2	['11', '12']	['9', '13']	['10', '10']	['12', '13']	['17', '17']	['9', '9']	['X', 'Y']	['8', '9']	['10', '11']
0.94(34/36)	CRL-11997	HEP-G2/2.2.1	['11', '12']	['9', '13']	['10', '10']	['12', '13']	['17', '17']	['9', '9']	['X', 'Y']	['8', '9']	['10', '11']
0.94(34/36)	HB-8065	Hep-G2	['11', '12']	['9', '13']	['10', '10']	['12', '13']	['17', '17']	['9', '9']	['X', 'Y']	['8', '9']	['10', '11']
0.94(34/36)	HB-8065.1	HepG2/SF	['11', '12']	['9', '13']	['10', '10']	['12', '13']	['17', '17']	['9', '9']	['X', 'Y']	['8', '9']	['10', '11']
0.94(34/36)	HPACC	Hep-G2	['11', '12']	['9', '13']	['10', '10']	['12', '13']	['17', '17']	['9', '9']	['X', 'Y']	['8', '9']	['10', '11']
0.94(34/36)	JCRB1054	Hep-G2	['11', '12']	['9', '13']	['10', '10']	['12', '13']	['17', '17']	['9', '9']	['X', 'Y']	['8', '9']	['10', '11']
0.94(34/36)	KCLB-Korea-88065	HepG2	['11', '12']	['9', '13']	['10', '10']	['12', '13']	['17', '17']	['9', '9']	['X', 'Y']	['8', '9']	['10', '11']
0.94(34/36)	RCB1648	Hep-G2	['11', '12']	['9', '13']	['10', '10']	['12', '13']	['17', '17']	['9', '9']	['X', 'Y']	['8', '9']	['10', '11']
0.94(34/36)	RCB1886	Hep-G2	['11', '12']	['9', '13']	['10', '10']	['12', '13']	['17', '17']	['9', '9']	['X', 'Y']	['8', '9']	['10', '11']
0.94(34/36)	CVCL_0027	Hep-G2	['11', '12']	['9', '13']	['10', '10']	['12', '13']	['17', '17']	['9', '9']	['X', 'Y']	['8', '9']	['10', '11']
0.94(34/36)	CVCL_3701	Hep-G2/2.2.1	['11', '12']	['9', '13']	['10', '10']	['12', '13']	['17', '17']	['9', '9']	['X', 'Y']	['8', '9']	['10', '11']
0.94(34/36)	CVCL_L855	Hep-G2/2.2.15	['11', '12']	['9', '13']	['10', '10']	['12', '13']	['17', '17']	['9', '9']	['X', 'Y']	['8', '9']	['10', '11']
0.94(34/36)	CVCL_AS98	Hep-G2/SF	['11', '12']	['9', '13']	['10', '10']	['12', '13']	['17', '17']	['9', '9']	['X', 'Y']	['8', '9']	['10', '11']
0.94(34/36)	CVCL_JG47	HepG2-Luc	['11', '12']	['9', '13']	['10', '10']	['12', '13']	['17', '17']	['9', '9']	['X', 'Y']	['8', '9']	['10', '11']
0.89(32/36)	CRL-10741	C3A-[HepG2/C3A,derivative-of-Hep-G2 (ATCC-HB-8065)]	['13', '11']	['13', '9']	['10', '10']	['13', '12']	['17', '17']	['9', '9']	['Y', 'X']	['9', '8']	['11', '10']
0.89(32/36)	CVCL_1098	Hep-G2/C3A	['11', '13']	['9', '13']	['10', '10']	['12', '13']	['17', '17']	['9', '9']	['X', 'Y']	['8', '9']	['10', '11']
0.83(30/36)	CVCL_A8FT	HepG2-R	['11', '12']	['9', '13']	['9', '10']	['12', '13']	['17', '17']	['9', '9']	['X', 'Y']	['8', '9']	['10', '12']
0.76(27/36)	CVCL_1T06	H7D7A	['nan']	['nan']	['10', '10']	['12', '13']	['17', '17']	['9', '9']	['X', 'Y']	['8', '9']	['10', '11']
0.76(27/36)	CVCL_1T07	H7D7B	['nan']	['nan']	['10', '10']	['12', '13']	['17', '17']	['9', '9']	['X', 'Y']	['8', '9']	['10', '11']
0.76(27/36)	CVCL_1T08	H7D7C	['nan']	['nan']	['10', '10']	['12', '13']	['17', '17']	['9', '9']	['X', 'Y']	['8', '9']	['10', '11']
0.76(27/36)	CVCL_1T09	H7D7D	['nan']	['nan']	['10', '10']	['12', '13']	['17', '17']	['9', '9']	['X', 'Y']	['8', '9']	['10', '11']
0.72(26/36)	CVCL_1312	IST-Mes2	['12', '13']	['13', '13']	['10', '10']	['11', '11']	['17', '17']	['9', '9']	['X', 'Y']	['8', '11']	['10', '11']
0.72(26/36)	CVCL_JG30	RM-P1	['9', '12']	['9', '14']	['10', '10']	['12', '12']	['17', '18']	['9', '9']	['X', 'X']	['8', '11']	['10', '11']
0.72(26/36)	CVCL_0465	OVCAR-3	['11', '12']	['12', '12']	['10', '10']	['12', '12']	['17', '17']	['9', '9']	['X', 'X']	['8', '8']	['11', '12']
0.67(24/36)	ACC-31	KARPAS-299	['11', '12']	['8', '12']	['10', '11']	['12', '12']	['17', '19']	['7', '9']	['X', 'Y']	['8', '8']	['10', '11']
0.67(24/36)	ACC-507	ONCO-DG-1	['11', '12']	['12', '12']	['10', '10']	['12', '12']	['17', '17']	['9', '9.3']	['X', 'X']	['8', '8']	['11', '12']
0.67(24/36)	ACC-537	ME-1	['11', '9']	['11', '9']	['11', '10']	['12', '12']	['17', '14']	['9', '9']	['Y', 'X']	['11', '8']	['12', '11']
0.67(24/36)	ATCC	OVCAR-3	['11', '12']	['12', '12']	['10', '10']	['12', '12']	['17', '17']	['9', '9.3']	['X', 'X']	['8', '8']	['11', '12']
0.67(24/36)	CRL-2061	SJCRH30 [RC13,RMS-13,SJRH30]	['12', '13']	['11', '11']	['10', '10']	['12', '12']	['17', '18']	['9', '9.3']	['X', 'Y']	['8', '11']	['10', '11']
0.67(24/36)	CRL-5826	NCI-H226 [H226]	['11', '12']	['13', '14']	['8', '10']	['9', '12']	['17', '17']	['8', '9.3']	['X', 'Y']	['8', '11']	['10', '11']
0.67(24/36)	CRL-7433	Hs-697.Sp	['11', '12']	['9', '11']	['10', '12']	['9', '12']	['17', '17']	['7', '8']	['X', 'Y']	['8', '9']	['11', '11']
0.67(24/36)	HPACC	CESS	['11', '12']	['12', '12']	['10', '12']	['12', '12']	['16', '17']	['7', '9.3']	['X', 'Y']	['8', '9']	['10', '11']
0.67(24/36)	HPACC	ZL5	['12', '12']	['9', '13']	['10', '11']	['12', '12']	['16', '17']	['6', '9.3']	['X', 'Y']	['8', '11']	['10', '11']
0.67(24/36)	HTB-161	NIH:OVCAR-3	['11', '12']	['12', '12']	['10', '10']	['12', '12']	['17', '17']	['9', '9.3']	['X', 'X']	['8', '8']	['11', '12']
0.67(24/36)	KCLB-Korea-30161	NIH:OVCAR-3	['11', '12']	['12', '12']	['10', '10']	['12', '12']	['17', '17']	['9', '9.3']	['X', 'X']	['8', '8']	['11', '12']
0.67(24/36)	RCB2135	NIH:OVCAR-3	['11', '12']	['12', '12']	['10', '10']	['12', '12']	['17', '17']	['9', '9.3']	['X', 'X']	['8', '8']	['11', '12']

细胞遗传质量鉴定检测

Cell Line Authentication Service

QPCR 检测报告

送检单位： 镜像绮点

检品名称： 细胞系

委托单位： 上海翼和应用生物技术有限公司

报告日期： 2024/05/28

Biowing Biotechnology, Shanghai, China 邮箱：zhusn@biowing.com.cn 技术服务咨询：86+18521538068
联系地址：上海市松江区广富林街龙腾路 1015 弄 2 号楼 518 室

报告说明

1. 本报告只对送检的来样负责。
2. 检验报告上的检验结果和检验单位名称，未经同意不得用于广告、评优及商业宣传。
3. 对本报告有异议，请于收到报告之日起十五日内以书面方式提出，逾期不予受理。
4. 对纸质检验报告涂改、增删，或未加盖检验单位印章的复印件均无效。

Biowing Biotechnology, Shanghai, China 邮箱：zhusn@biowing.com.cn 技术服务咨询：86+18521538068
联系地址：上海市松江区广富林街龙腾路 1015 弄 2 号楼 518 室

样 品 信 息

样品编号：

客户样本编号	**公司编号**
hepG2	20240527-01

样品数量： 1

样品性状： 细胞系

检测项目： 种属鉴定

检测方法： 用 Axygen 的基因组抽提试剂盒提取 DNA，采用多重 PCR 体系，人源、小鼠、大鼠、仓鼠四色荧光探针 QPCR 扩增方案扩增，在实时荧光定量检测仪进行检测。

Biowing Biotechnology, Shanghai, China 邮箱：zhusn@biowing.com.cn 技术服务咨询：86+18521538068
联系地址：上海市松江区广富林街龙腾路 1015 弄 2 号楼 518 室

检 测 结 果

(一)　检验基本情况

编号	客户样本编号	匹配种属
20240527-01	hepG2	人源

(二)　结果描述

20240527-01：该细胞存**人源**细胞 DNA，该株细胞鉴定结果为**人源细胞系**，本次检测在该细胞系中**无大鼠污染，无仓鼠污染，无小鼠污染**。。

微量紫外分光光度计测定 DNA 质量，结果如下表所示

编号	浓度(ng/μL)	A260	A280	260/280	260/230
20240527-01	315.26	6.305	3.122	2.02	1.91

（三）QPCR 检测结果

1.检测结果汇总表

编号	Human-CT	Mouse-CT	Hamster-CT	Rat-CT
20240527-01	20.66	undet	undet	undet
阳性参考品	23.64	22.90	22.48	22.01
阴性参考品	undet	undet	undet	undet

Biowing Biotechnology, Shanghai, China 邮箱：zhusn@biowing.com.cn 技术服务咨询：86+18521538068
联系地址：上海市松江区广富林街龙腾路 1015 弄 2 号楼 518 室

2. 样品和对照扩增曲线

20240527-01：

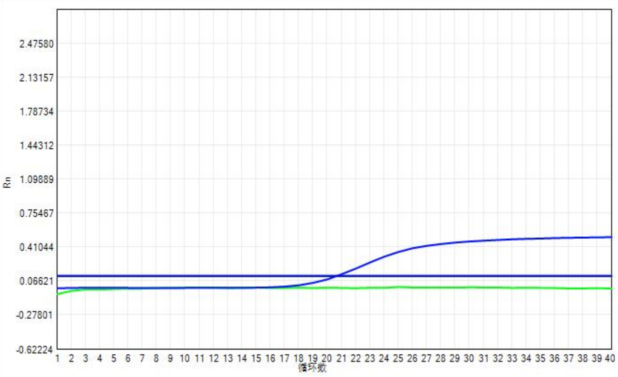

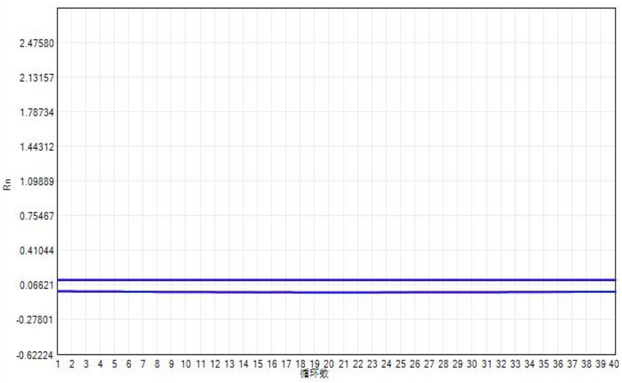

样品 qPCR 检测结果

注： 检测通道 1：Mouse (HEX 绿), Human (FAM1 蓝)； 检测通道 2：Hamster(FAM2 蓝), Rat (CY5 红)

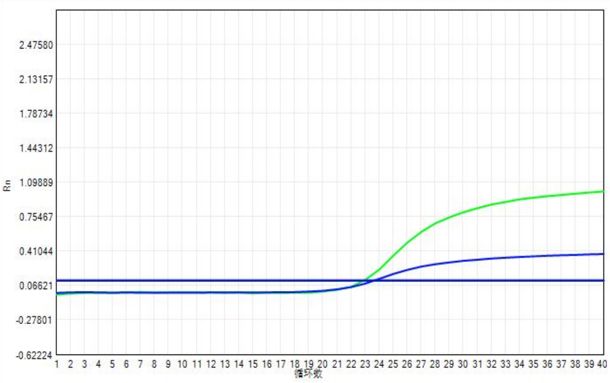

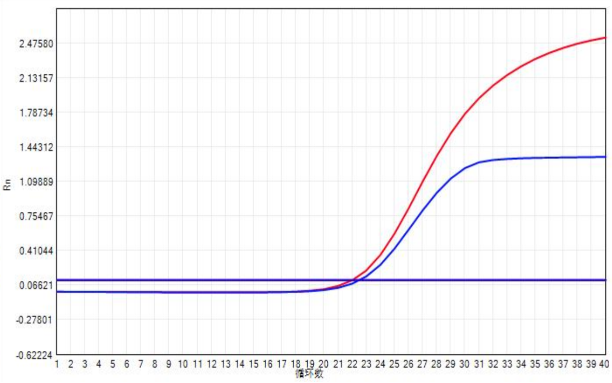

阳性对照 qPCR 检测结果

注： 阳性对照通道 1：Mouse (HEX 绿), Human (FAM1 蓝)； 对照通道 2：Hamster(FAM2 蓝), Rat (CY5 红)

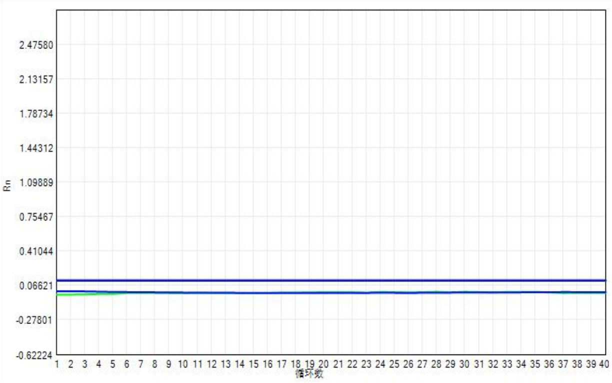

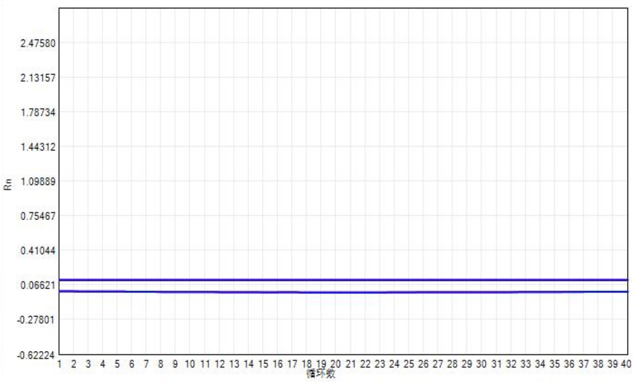

阴性对照 qPCR 检测结果

注： 阴性对照通道 1：Mouse (HEX 绿), Human (FAM1 蓝)； 对照通道 2：Hamster(FAM2 蓝), Rat (CY5 红)

其他说明

(一) 分型方案及位点分布

	检测通道	对照通道	阴性通道
1	Human (FAM1)<br>Mouse (HEX)	Human (FAM1)<br>Mouse (HEX)	Human (FAM1)<br>Mouse (HEX)
2	Hamster (FAM2)<br>Rat(CY5)	Hamster(FAM2)<br>Rat(CY5)	Hamster (FAM2)<br>Rat(CY5)

(二) 结果判定

模板类型	Ct 值	判定结果
阳性对照	22±2	阳性成立
	＞25	阳性不成立
阴性对照	＞35	阴性成立
	＜35	阴性不成立

主要实验人员： 胡红瑞

复核人: 陈婷

负责人： 巢凯悦

签发日期： 2024/05/28

Biowing Biotechnology, Shanghai, China 邮箱：zhusn@biowing.com.cn 技术服务咨询：86+18521538068
联系地址：上海市松江区广富林街龙腾路 1015 弄 2 号楼 518 室
