# Supplementary material for: Multifunctional saikosaponin D-liposomes for hepatocellular carcinoma: Formulation optimization, characterization, and in vitro/in vivo evaluation
Source: Int J Pharm X. 2025 Nov 11;10:100445. doi: 10.1016/j.ijpx.2025.100445 (PMC12664412; doi:10.1016/j.ijpx.2025.100445)
Supplement: Supplementary file 4 — Supplementary material 4 [file mmc4.pdf]

Report of Cell Line Authentication

（Notice:This authentication report is restricted to the cell sold from Guangzhou Cellcook Biotech Co., Ltd, and the date with seal is the date of delivery. ）

Ⅰ. Sample

Sample Name: labeled as ‘H22’.

Ⅱ. Method and Procedure

1. Amplification of gene COX1 and electrophoresis are employed to survey the species of the sample.

Ⅲ. Results

H22: ①The sample is a mouse (*Mus musculus*) cell line. No other species (*Homo sapiens, Cricetulus griseus, Rattus norvegicus, Macaca mulatta, Cercopithecus aethiops, Canis familiaris, Bos Taurus*, IC) cells were found in the sample.

Operator: Xiaohua Mo

Auditor: Xuanyi Liang

Guangzhou Cellcook Biotech Co., Ltd

GUANGZHOU CELLCOOK BIOTECH CO.,LTD
16 Mar 2023
CELL STR PROFILING

Figure 1. Authentication of the species of the sample

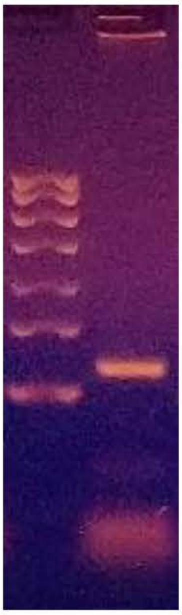

M: Marker. As the size of 700, 600, 500, 400, 300, 200 and 100bp from up to down.

Nine species are checked, as follow: *Homo sapiens* 391bp, *Cricetulus griseus*315bp, *Macaca mulatta*287bp, *Cercopithecus aethiops*222bp, *Rattus norvegicus*196bp, *Canis familiaris*172bp, *Mus musculus*150bp, *Bos Taurus*102bp, IC 70bp

The sample. The band size is 150bp which matches the size of *Mus musculus*.
